# A universal Fab targeting a conserved U1A–RNA epitope for RNA structure determination by cryo-EM

**DOI:** 10.1093/nar/gkag502

**Published:** 2026-05-20

**Authors:** Ekaterina V Filippova, Daniel Krochmal, Somnath Mukherjee, Joseph A Piccirilli, Anthony A Kossiakoff

**Affiliations:** Department of Biochemistry and Molecular Biology, University of Chicago, Chicago, IL 60637, United States; Department of Biochemistry and Molecular Biology, University of Chicago, Chicago, IL 60637, United States; Department of Biochemistry and Molecular Biology, University of Chicago, Chicago, IL 60637, United States; Department of Biochemistry and Molecular Biology, University of Chicago, Chicago, IL 60637, United States; Department of Chemistry, University of Chicago, Chicago, IL 60637, United States; Department of Biochemistry and Molecular Biology, University of Chicago, Chicago, IL 60637, United States

## Abstract

Recent advances in cryo-electron microscopy (cryo-EM) have made antigen-binding fragments (Fabs) essential tools in the field of structural biology. Fabs facilitate image alignment, thereby enhancing three-dimensional (3D) reconstruction, and increase the effective size of proteins, aiding in their structural elucidation. In this study, we sought to broaden the use of Fabs as fiducial markers to elucidate the structures of RNA molecules. Identifying an appropriate Fab for a specific RNA target can be particularly challenging due to RNA’s inherent flexibility and tendency to assume multiple conformations, which complicate the process and prolong the structure determination timeline. To address this challenge, we designed a universal Fab that specifically recognizes a U1A–RNA epitope, thereby reducing the need for Fab selection tailored to each individual RNA target. We determined the cryo-EM structure of the class I ligase ribozyme complexed with a portable U1hpII loop bound to the U1A protein and the Fab. The resulting structure revealed that the Fab interacts with a conserved U1A–RNA binding region, which can be engineered into other RNA molecules. This strategy presents significant potential for streamlining the structural determination of various RNAs, which are essential for biological and biomedical research.

## Graphical Abstract

**Graphical Abstract:**
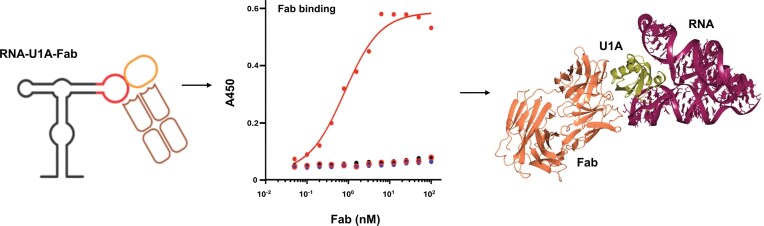

## Introduction

Cryo-electron microscopy (Cryo-EM) has become a powerful methodology, allowing direct visualization of macromolecules in solution and enabling high-resolution analysis of large macromolecular complexes in various conformational states. Despite recent advances in single-particle cryo-electron microscopy (SPA cryo-EM), obtaining high-resolution structures of RNA molecules remains difficult. RNAs often exhibit significant conformational heterogeneity due to their inherent flexibility, influenced by their secondary and tertiary structures, the torsional and hydrogen-bonding effects of the 2′-hydroxyl group, and their strong reliance on the surrounding ionic environment. While this dynamic behavior is often crucial for RNA function, it makes particle alignment and reconstruction in single-particle analysis more challenging.

As a result, new approaches are being developed to increase the success rate of RNA structure determination. One such method involves engineering large, symmetrical RNA oligomers. This technique, called “ROCK” (RNA oligomerization-enabled cryo-EM via installing kissing loops), encourages the self-assembly of RNA into ring-shaped symmetrical oligomers by inserting kissing stem-loops into non-functional regions of the RNA structure [1]. Complementing this strategy, recent advances in RNA structural biology have introduced additional scaffolding approaches, including the design of 2- and 4-fold symmetric architectures that enable *de novo* structure determination of covalently attached RNA [2], as well as a method that fuse small RNAs into a group II intron [3]. Additionally, combining SPA cryo-EM, X-ray crystallography, and NMR spectroscopy with computational modeling tools, such as *auto-DRRAFTER*, accelerates RNA modeling and aids in structure elucidation [4, 5].

Natural and synthetic RNA-binding proteins (RBPs), such as U1 small nuclear ribonucleoprotein A (U1A), the ribosomal protein L7Ae, host factor-I protein (Hfq), and synthetic anti-RNA antibody fragments (Fabs), have significantly advanced RNA structural studies utilizing techniques such as X-ray crystallography and SPA cryo-EM [6–24]. Employing RNA-binding proteins is particularly beneficial for examining RNA–protein interactions or conformational alterations in RNA induced by these interactions. For instance, conjugating a Fab fragment to a small RNA, such as a riboswitch, has facilitated high-resolution crystallography to elucidate the ligand-binding site and related conformational changes in the RNA [23, 25, 26]. Although numerous RNA–Fab complex structures have been resolved by X-ray crystallography, cryo-EM studies predominantly involve large ribonucleoprotein complexes. Simultaneously, Fabs are extensively utilized in cryo-EM to determine the structures of membrane proteins [27, 28]. Fundamentally, the combination of Fabs with SPA cryo-EM presents several advantages for RNA structural investigations: (i) the addition of approximately 50 kDa Fab increases particle mass and enhances RNA visibility; (ii) Fab binding supplies fiducial markers for particle alignment; (iii) Fabs can be engineered against a variety of RNA targets; and (iv) this methodology has the potential to capture multiple RNA conformations, which may be critical for RNA functionality. Nevertheless, selecting appropriate Fabs for RNA targets remains challenging and time-consuming due to RNA’s inherent flexibility and the limited availability of phage-display libraries targeting RNA or nucleotide fragments.

To address these challenges, we aimed to develop a Fab that targets a conserved U1A–RNA binding motif and can be adapted to various RNA structures, thereby removing the need to generate Fabs for each specific RNA. In this study, we present a strategy for creating a Fab that can be applied to any RNA after inserting a well-characterized U1hpII loop, which serves as the cognate binding site for the spliceosomal protein U1A [29]. It has been demonstrated that engineered RNA containing the U1hpII loop forms a highly stable RNA–U1A complex without causing conformational rearrangements in the studied RNA structures [6, 7, 9, 10, 26]. To validate the selected Fab as a universal fiducial marker, we determined the cryo-EM structure of the U1A–Fab complex with the class I ligase (cIL) ribozyme in the presence of the secondary fiducial sfFab18–PGA1 at a global resolution of 3.04 Å. The sfFab18–PGA1 construct was used to enhance particle visibility and served as a supplementary tool for cryo-EM analysis of the primary cIL–U1A–Fab1R complex. The determined structure explains the chemical and structural basis for the Fab’s exceptional specificity to the conserved region involving U1A and the U1hpII loop, making it a valuable tool for cryo-EM structure determination of diverse RNAs. We believe this innovative approach offers an opportunity to simplify structural analysis across a wide range of RNAs, paving the way for significant advancements in biological and biomedical research.

## Materials and methods

### RNA preparation

*In vitro* transcription reactions were performed using DNA templates prepared by PCR amplification of single-stranded DNA oligomers with T7 promoter sequence purchased from Integrated DNA Technologies. The reverse primer contained 2′-*O*-methyl modifications in the first two nucleotides to reduce heterogeneity at the 3′ end of transcripts [30]. RNA was prepared by transcription for 3 h at 37°C in buffer containing 40 mM Tris–HCl pH 8.0, 2 mM spermidine, 10 mM NaCl, 25 mM MgCl_2_, 10 mM dithiothreitol (DTT), 40 U/ml Rnase inhibitor (QIAGEN), 5 U/ml thermostable inorganic pyrophosphatase (TIPPase, New England Biolabs), 5 mM of each nucleoside triphosphate, 100 pmol/ml DNA template, and 50 μg/ml homemade T7 RNA polymerase [31]. Transcription reactions were quenched by adding 5 U/ml RNase-free DNase I (Promega) and incubating at 37°C for 30 min. Following DNase treatment, RNA was purified by denaturing polyacrylamide gel electrophoresis (urea–PAGE). The RNA band was visualized by UV shadowing, excised from the gel, and eluted overnight at 4°C in 10 mM Tris pH 8.0, 2 mM ethylenediaminetetraacetic acid (EDTA), and 300 mM NaCl buffer. The buffer for eluted RNA was exchanged three times for pure water using a 10-kDa cutoff size-exclusion column (Amicon). RNA was collected, aliquoted and stored at −80°C until further use.

### RNA refolding

An aliquot of RNA was diluted in nuclease-free water, heated at 95°C for 2 min to denature the RNA, and then snap-cooled at room temperature (RT) for 5 min. The RNA was subsequently incubated in a buffer containing 10 mM Tris pH 7.5, 100 mM KCl, 5 mM MgCl_2_, 0.05% Tween-20 for Spinach RNA, and 20 mM HEPES pH 7.4, 150 mM NaCl, 10 mM MgCl_2_, 0.05% Tween-20 for cIL, and U1hpII at 50°C for 10 minutes. It was then cooled for an additional 5 min at RT, preparing it for experiments. For cryo-EM SPA, the cIL RNA was refolded in 10 mM HEPES pH 7.4, 75 mM NaCl, and 5 mM MgCl_2_.

### Phage display selection

The U1hpII–U1A complex for the selection was prepared by mixing the equimolar amounts of biotinylated U1hpII RNA and U1A or U1hpII RNA and biotinylated SNAP fused to U1A with a linker containing thrombin cleavage site, followed by incubation for 30 min at RT. The selection campaign was performed using library E at RT, following published procedures [32].

In the first round, 1 ml of phage library E [33], containing 10¹²–10¹³ virions, was pre-incubated with 2 µM SNAP in PBST/0.1% BSA for 30 min to pre-clear the library. In parallel, 250 µl of streptavidin (SA) magnetic beads (Promega) were precoated with 200 nM biotinylated target and then blocked with 5 µM D-biotin to saturate unoccupied SA sites and prevent non-specific phage binding. The pre-cleared phage pool was added to the prepared beads and incubated for 30 min. Beads with bound virions were washed extensively with a buffer containing PBST/0.1% BSA, 10 mM MgCl₂, 0.4 U/µl murine RNase inhibitor (NEB), and 1 µM SNAP. These beads were then used to infect freshly grown log-phase *Escherichia coli* XL1-Blue cells. Phages were amplified overnight in 2YT media with 50 μg/ml ampicillin and 10^9^ plaque-forming units/ml of M13KO7 helper phage. To increase the selection stringency, three additional rounds of sorting were performed by decreasing the target concentration in each round: second round − 200 nM; third round − 50 nM; fourth round − 10 nM, using the amplified virion pool from the previous round as input. Sorting from the second to the fourth rounds was done on a Kingfisher instrument with the phage pool premixed with the biotinylated target for 20 min before immobilization on the beads. From the second round onward, RNase A (Thermo Scientific, 1 µg/ml, 10 min) or thrombin (Novagen, 1 U/ml, 10 min) was used to elute the bound phage from the target immobilized via biotinylated U1hpII or SNAP–U1A, respectively. After four rounds of selection, individual clones were evaluated using single-point phage ELISA, and those displaying a high signal-to-noise ratio and specific binding to the U1hpII–U1A complex were sequenced to identify unique binders. The most dominant binders were then subcloned into vector pSFV4, expressed in *E. coli* BL21 cells, and purified following published protocols [34].

A phage pool consisting of the unique binders identified from the initial selection was used as input to select for binders with slower off-rates. In this strategy, a large excess of non-biotinylated complex was used as a competitor to deplete the phage pool of binders with fast off-rates. Two rounds of selection were performed on the Kingfisher instrument. In the first round, the phage pool was incubated for 15 min with 1 nM biotinylated SNAP–U1A:U1hpII complex, followed by a 15-min incubation with 1 µM non-biotinylated complex to eliminate binders with faster off-rates. In the second round, the concentration of the biotinylated complex was reduced to 0.1 nM, and incubation time with the non-biotinylated complex was extended to 45 min to further enrich for binders with very slow off-rates. The unique binders obtained after two rounds of competitive selection were cloned into the vector pSFV4, expressed, and purified.

### ELISA experiments

All ELISA experiments were conducted using a 96-well plate coated with 50 μl of 2 μg/ml neutravidin in Na_2_CO_3_/NaHCO_3_ buffer at pH 9.6, followed by blocking with 1% BSA in PBS. A single-point phage ELISA was used to quickly screen the binding of Fabs obtained after selection in phage format. Colonies of *E. coli* XL1-Blue harboring phagemids were directly inoculated into 400 μl of 2YT broth supplemented with 100 μg/ml ampicillin and M13KO7 helper phage. The cultures grew at 37°C for 16–20 h in a 96-deep-well plate. Culture supernatants containing Fab phage were diluted 10-fold in PBS with 0.05% Tween-20 and 10 mM MgCl_2_ buffer, then transferred to Maxisorp plates (Thermo Scientific^™^) pre-coated with 20 nM biotinylated targets in experimental wells, and buffer in control wells, and incubated for 30 min at RT . The ELISA plates were incubated with the phage for 20 min, washed with PBS containing 0.05% Tween-20 and 10 mM MgCl_2_, then incubated with HRP-conjugated anti-M13 mouse monoclonal antibody (diluted 1:5000 in PBST) for 30 min. After washing again, the plates were developed with TMB/H_2_O_2_ (Thermo Scientific) and quenched with 10% H_3_PO_4_. Absorbance at 450 nm was measured. The background binding of the phage was assessed by the absorbance from the control wells.

Protein-based multipoint ELISA was conducted to evaluate the affinity and specificity of the generated Fabs to the U1hpII–U1A complex. A 20 nM concentration of biotinylated U1hpII–U1A complex was immobilized on a neutravidin-coated, BSA-blocked ELISA plate, followed by a 20-min incubation with 2-fold serial dilutions of the purified Fabs starting at 100 nM. The plates were washed, and the bound antigen–Fab complexes were incubated with a secondary HRP-conjugated anti-human F(ab’)2 monoclonal antibody (diluted 1:5000 in PBST). Similar to phage ELISA, the plates were washed again, developed with TMB/H_2_O_2_, quenched with 10% H_3_PO_4_, and the absorbance at 450 nm (A450) was measured. To determine the affinity, the data were fitted with a non-linear sigmoidal curve with a variable slope using GraphPad PRISM, and the EC50 value was calculated.

### Binding kinetics with surface plasmon resonance

All SPR experiments were conducted at 20°C using a MASS-1 (Bruker) instrument and high-affinity streptavidin (SA) sensor chips. The target RNAs were co-transcriptionally tagged with a 22-nt 3′ end overhang to enable reversible immobilization on the SA sensor chip surface via a biotinylated complementary DNA sequence. For the SPR experiments, the RNA was refolded as described above. To measure the RNA–U1A binding kinetics, the refolded RNA, diluted in the appropriate folding buffer, was injected onto the chip surface activated with a biotinylated DNA handle. Subsequently, 2-fold serial dilutions of U1A were injected, and the association and dissociation phases were recorded. After each dilution measurement, the chip surface was regenerated with 50 mM NaOH/1 M NaCl and re-equilibrated with the running buffer to prepare for the next cycle. For each kinetic assay, at least four dilutions of the analyte were tested.

To measure the Fab binding to the RNA–U1A complex, the RNA was incubated with an equimolar amount of U1A for 30 min at RT following the refolding. The sensograms were processed and analyzed using MASS-1 Evaluation Software (Bruker). The *k*_on_ and *k*_off_ values were determined using a 1:1 Langmuir binding model by fitting an exponential association function to the association phase and an exponential decay function to the dissociation phase.

### Expression and purification of Fabs

The amplified gene encoding the heavy and light chains of the selected Fabs was cloned into the expression vector pSFV4. Additionally, the LRT mutation was introduced into the light chain of Fab1R to increase its affinity for PGA1 (an engineered variant of wt protein G) for structural studies [35]. All Fabs were expressed and purified using established protocols [36]. sfFab18 (secondary fiducial) was expressed and purified in the same manner.

### Expression and purification of U1A and PGA1

The gene encoding the N-terminal RRM-I domain of the U1A Y31H/Q36R double mutant protein from the spliceosomal U1 nuclear ribonucleoprotein (U1snRNP) complex in *Homo sapiens* (residues 1–101) was PCR-amplified and cloned into the IPTG-inducible, kanamycin-resistant pHFT2 vector. This vector contains an N-terminal 10xHis affinity tag and a TEV protease cleavage site. *E. coli* BL21(DE3) cells harboring the U1A plasmid were grown in TB medium to an OD600 of 0.6 at 37°C, then induced with 1 mM IPTG at 19°C overnight (250 rpm). After incubation, the cells were harvested by centrifugation at 6000 rpm for 10 min at 19°C and lysed by sonication in a buffer containing 20 mM Tris–HCl, 500 mM NaCl, 10% (w/v) glycerol, 10 mM imidazole, and 1 mM phenylmethylsulfonyl fluoride (PMSF) at pH 8.0. Cell debris was removed by centrifugation at 23 000 rpm for 1 h at 4°C.

U1A was purified using His-tag affinity chromatography with a 5 ml of Cytiva HisTrap FF column (GE Healthcare). The purification involved washing with a buffer containing 20 mM Tris–HCl, 500 mM NaCl, and 10% (w/v) glycerol at pH 8.0. U1A was then eluted using a linear gradient of 500 mM imidazole in the same buffer. The eluted U1A was mixed with TEV protease at a 1:20 molar ratio and dialyzed overnight at 4°C in a buffer with 50 mM HEPES (pH 7.8), 500 mM NaCl, 5% (w/v) glycerol, 5 mM EDTA, and 5 mM β-mercaptoethanol (BME) to remove the His-tag. To remove EDTA, the U1A-TEV solution was dialyzed overnight at 4°C in a loading buffer with 50 mM HEPES (pH 7.8), 500 mM NaCl, and 5% (w/v) glycerol. Finally, His-tag-free U1A was separated on a 5 ml of Ni-NTA column pre-equilibrated with the loading HEPES buffer.

U1A was further purified by size exclusion chromatography on a Superdex 200 Increase 10/300 GL column using the loading buffer. Next, it was purified on a 5 mL CHT-I hydroxyapatite column (Bio-Rad) with gradient elution in a buffer containing 10 mM K_2_SO_4_ and 50 mM KCl at pH 7.8. U1A was eluted using 500 mM (NH_4_)_2_SO_4_. Finally, U1A was dialyzed in a buffer containing 20 mM HEPES (pH 8.0), 500 mM NaCl, and 10% (w/v) glycerol.

All purification steps were performed at 4°C using the ÄKTAxpress^™^ (GE Healthcare Life Sciences) high-throughput purification system, which was pre-washed with 0.5 M sodium hydroxide and nuclease-free water to eliminate any nucleases. The final purity of U1A was assessed by SDS–PAGE.

PGA1 (engineered mutant of wild-type PG [37]) was cloned into the pHFT2 vector, expressed in *E. coli* BL21(DE3) cells, and purified using Ni-affinity and size-exclusion chromatography following a protocol similar to that used for U1A protein.

### Preparation of RNA–U1A–Fab1R–PGA1–sfFab18 complex

Before forming complexes, all purified proteins were checked for RNase contamination using the RNase Alert Lab Test Kit (Thermo Fisher Scientific). U1A, Fab1R, PGA1, sfFab18, and refolded cIL RNA were mixed in a 1:1:1:1:1.2 molar ratio in loading buffer containing 10 mM HEPES, 75 mM NaCl, 5 mM MgCl_2_ at pH 7.4. The proteins and RNA were incubated on ice for 30 min before being loaded onto a Superdex 200 Increase 10/300 GL column pre-equilibrated with the same buffer. The eluted fraction containing the monodisperse complex with all components was collected (Supplementary Fig. S4A) and analyzed by SDS–PAGE and urea–PAGE to evaluate the integrity of the proteins and RNA, respectively.

### Sample preparation and cryo-EM data collection

The complex was applied to glow-discharged grids, which were then blotted and rapidly plunged into liquid ethane using a Vitrobot Mark IV (Thermo Fisher Scientific). For cryo-EM analysis, the complex was imaged on a Titan Krios G3i cryo-electron microscope (Thermo Fisher Scientific) equipped with an energy filter (BioQuantum) and Gatan K3 electron detector operating at 300 kV at the University of Chicago Advanced Electron Microscopy Facility (Chicago, IL). Automated data collection was performed using EPU software (Thermo Fisher Scientific). A total of 10 696 movies were collected for the cIL–U1A–Fab1R–PGA1–sfFab18 structure −6080 from the first data set and 4616 from the second. All movies were collected with a super-resolution pixel size of 0.534 Å and a defocus range of −0.9 to −2.5 μm at a nominal magnification of 81 000. For the first and second data sets, movies consisted of 52 and 50 frames, respectively, with total exposures of 60 e⁻/Å² and total exposure times of 7.1 and 7.2 s. Detailed sample preparation and data collection parameters for the complex are provided in Supplementary Table S3. A representative motion-corrected cryo-EM micrograph of the cIL–U1A–Fab1R–PGA1–sfFab18 complex is shown in Supplementary Fig. S4B.

### Single-particle cryo-EM image processing and map reconstruction

The standard *cryoSPARC* workflow [38] was followed to obtain the reconstruction of the complex. Each dataset was processed independently, including patch-based motion correction and patch CTF estimation. During exposure curation, manual inspection led to the exclusion of 217 and 640 movies from the first and second datasets, respectively, due to ice defects, broken holes, contamination, drift, or poor CTF fits. Initial particle picking was performed on a small subset of movies, and 2D classification of the extracted particles was used to generate templates for template-based picking, with a total of 327 742 particles contributing to the initial templates. Particles extracted from all remaining movies across both datasets were combined and subjected to multiple rounds of 2D classification to remove poor-quality classes. A total of 1 264 747 particles displaying well-resolved features were then used for two consecutive rounds of *ab-initio* reconstruction and heterogeneous refinement with four classes. The class exhibiting a clear map of the complex was refined using non-uniform refinement and subsequently sharpened in *cryoSPARC* [38].

To improve the resolution of the core and RNA regions, a tight mask covering these regions was created in *Chimera* [39]. The core region was further refined using *cryoSPARC*, while the RNA mask, along with the map and particles from the non-uniform refinement, was exported to *RELION* [40] for 3D classification. The class containing 59 244 particles with well-resolved RNA structural features was refined in *cryoSPARC*. The resulting focused map of the RNA region was B-factor-sharpened in *cryoSPARC* for subsequent model building in *auto-DRRAFTER* [5]. The final focused maps corresponding to the RNA and core regions were generated using *phenix.map_box* in *Phenix* [41], with the refined structural models applied to optimize map visualization. Because focused refinement enhances local map quality while excluding density outside the selected region, a composite cryo-EM map of the entire cIL–U1A–Fab1R–PGA1–sfFab18VD complex was generated by combining the two focused maps using *phenix.combine_focused_maps* in *Phenix* [41]. This approach enabled integration of the highest-quality density from each locally refined region into a single map, allowing visualization of the complete assembly within a unified framework. The resulting composite map was subsequently processed with *phenix.map_box* using the combined structural model containing both the core and RNA regions to generate the final map showing the full complex. Details of the single-particle cryo-EM data processing for the complex are provided in Supplementary Table S3 and Supplementary Fig. S5. The resolution of the final maps was estimated using the 0.143 FSC criterion in *cryoSPARC* (reported resolutions correspond to the original consensus and focused refinements).

### Structural model building and refinement

The crystal structures of U1A:U1hpII (PDB ID: 3HHN), PGA1 (PDB ID: 6U8C), and Fab (PDB ID: 5BJZ) were initially fitted into the electron density map of the core region manually in *Chimera* using the “*fit-in-map*” tool for subsequent refinement [39]. The RNA–U1hpII model was built into the B-factor-sharpened RNA density map generated in *cryoSPARC* using *auto-DRRAFTER* [5] in two rounds, combining fully automated and semi-automated modes. In the first modeling round, fully automated mode was employed with the cIL sequence and secondary structure, resulting in 7500 initial models. The top 10 models converged with a mean pairwise RMSD of 10.5 Å (Supplementary Fig. S9A). The best-fitting model, which showed good agreement with the density map, was selected by visual inspection. This model aligned well with the cIL crystal structure, especially in the catalytic RNA domains (P3–P6–P7 and P4–P5). In the next modeling round, semi-automated mode was used, starting from the selected first-round model where the catalytic domains, including the U1hpII loop, were replaced with those from the RNA crystal structure (PDB ID: 3HHN). Semi-automated modeling generated 2000 models, with the top 10 converging to a mean RMSD of 3.1 Å. The models aligned well across all three domains, with the highest RMSD seen in the J1/3 region (Supplementary Fig. S9B and C). The final RNA structural model was based on the median model with the J1/3 region removed (Supplementary Fig. S9D). The structures of the core complex and RNA were then manually adjusted and refined in *Coot* [42], followed by optimization and automatic refinement of atomic coordinates using *phenix.real_space_refine* [43]. The RNA structure was further refined with *ERRASER* [44] and *Phenix* [41, 43]. The final models were validated using *MolProbity* [45]. The fit between the map and the model was assessed through cross-correlation coefficients (Supplementary Fig. S7). Figures were prepared using *Chimera* [39], *CCP4MG* [46], and *PyMOL* (Schrödinger, LLC).

## Results

### Design of RNA–U1A–Fab module for structural studies

**Figure 1.**
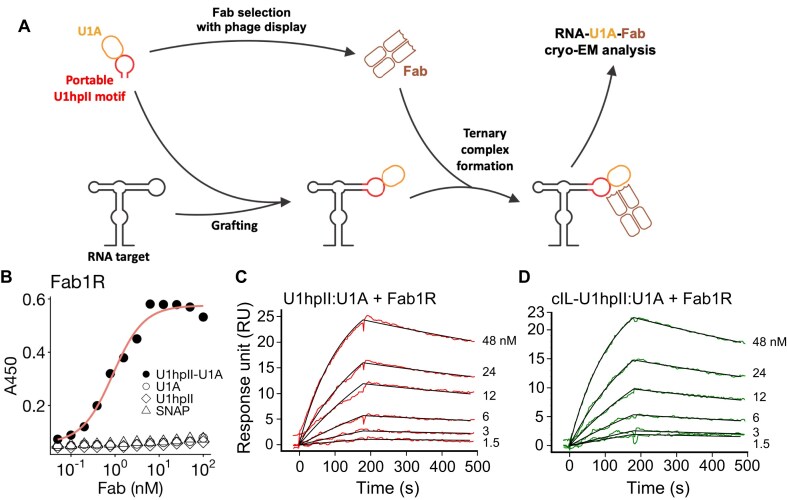
Generation and characterization of the U1hpII:U1A–Fab module. (**A**) Schematic overview of the development of the U1hpII:U1A–Fab module for SPA cryo-EM of RNA structures. (**B**) ELISA binding curves demonstrating specific recognition of the U1hpII:U1A complex by Fab1R. The curves for U1hpII:U1A are fitted with a non-linear sigmoidal function with variable slope for EC50 estimation. Fab1R shows no detectable binding to U1A alone, U1hpII alone, or SNAP. Binding responses are shown as absorbance at 450 nm plotted as a function of Fab concentration. (**C** and **D**) Surface plasmon resonance sensorgrams showing kinetic analysis of Fab1R binding to the U1hpII:U1A complex and the cIL–U1hpII:U1A complex, respectively. The concentrations of analyte injected are indicated. The data demonstrate that Fab1R retains high affinity for the U1hpII:U1A complex in the context of the larger RNA construct.

To facilitate cryo-EM RNA structure determination and minimize the need for Fab selection experiments for each individual RNA target, we aimed to develop a Fab-based fiducial mark that targets a conserved surface within RBP–RNA complexes. The scheme for creating this fiducial marker is outlined in Fig. 1A. For our study, we selected the N-terminal RRM domain of the U1A protein complexed with RNA containing an engineered high-affinity U1A binding site, known as hairpin II of the U1 snRNA (U1hpII) [47]. The interactions between U1A and U1hpII are well-characterized biochemically and it has been demonstrated that U1A binds with high specificity and affinity to the AUUGCAC loop [48]. The U1A-binding site can be engineered into the stem-loop region of diverse RNAs, enabling formation of a stable U1A–RNA complex. This strategy has been used effectively with the U1A:U1hpII module as a crystallization scaffold to facilitate high-resolution structural determination of numerous RNA molecules [6–10, 26, 47]. Therefore, we conjectured that Fabs against the U1A:U1hpII complex can be used as “plug and play” fiducial marks for structure determination of different RNAs once the U1hpII motif has been grafted successfully into the RNAs of interest. The post-ligation form of a well-studied artificial cIL ribozyme that facilitates the formation of phosphodiester bonds between RNA nucleotides [9, 26] was chosen as a model target for cryo-EM structure determination using these universal Fabs as fiducial marks.

### Generation and characterization of Fab against U1hpII:U1A module

To confirm the portability of the U1hpII:U1A module and its usability for cryo-EM, we measured the kinetics of the U1A:U1hpII stem-loop interaction within the context of other RNAs. Specifically, we replaced the apical loop of Spinach RNA [14, 16] and the L5 loop of cIL ribozyme [26] with the U1A-binding loop of U1hpII. The binding of U1A to these chimeric constructs was modestly affected (Supplementary Fig. S1 and Supplementary Table S1). However, the changes in *K*_D_ and *k*_off_ were not deemed significant enough to prevent its use in cryo-EM.

To generate a new Fab for the U1hpII:U1A complex, phage display selections were performed using a reduced genetic code phage display library E [33]. Initially, four rounds of biopanning were conducted with the complex immobilized on streptavidin-coated beads via biotinylated U1hpII or biotinylated SNAP-tagged U1A. Each round used progressively lower concentrations of the target complex. The selection process included soluble competitors such as BSA and SNAP, as well as Spinach RNA aptamer, to minimize the chance of selecting non-specific RNA-only binders. Additionally, selection was performed using biotinylated SNAP–U1A to identify U1A-only binders. After four rounds, 48 unique Fabs demonstrating specific binding to the U1hpII:U1A complex in single-point phage ELISA were identified. To select the top binders with the most favorable off-rate kinetics for cryo-EM analysis, all selected phage clones displaying the Fabs were combined in equal amounts and subjected to two additional rounds of competitive selection under increased pressure on the off-rate. The final round, performed at an antigen concentration of 0.1 nM, yielded a tenfold enrichment of phage over SNAP-only binders. This process resulted in a final pool of nine binders, of which eight were confirmed to be specific for the U1hpII:U1A complex (Fig. 1B and Supplementary Fig. S2). As shown by multipoint ELISA, these Fabs bind with high affinity to the complex, but do not bind to either component alone.

To evaluate the binding kinetics of selected Fabs to U1hpII:U1A, we employed SPR and DNA-directed RNA immobilization [49]. In this method, a roughly 20-nucleotide 3′ RNA overhang was co-transcriptionally added to the RNA target, allowing immobilization on the SA chip through hybridization with a biotinylated complementary DNA. These binders exhibited a range of affinities from 2 to 45 nM and off-rates between 6.6 × 10^−4^ and 3.3 × 10^−2^ s^−1^ (Fig. 1C, Supplementary Fig. S3, and Supplementary Table S2). Notably, the *k*_off_ values aligned with sequence enrichment after off-rate selection, binders with the slowest off-rates were highly enriched, forming a larger portion of the phage pool (Supplementary Table S2). Among these, Fab1R showed the highest enrichment and the most favorable binding properties, specifically recognizing the U1hpII:U1A complex with a *K*_D_ of 4.0 nM and a *k*_off_ of 6.6 × 10^−4^ s^−1^ (Fig. 1B and C, and Supplementary Table S2). Importantly, Fab1R maintained binding to the U1hpII:U1A epitope without negatively affecting the kinetics (*K*_D_ of 4.5 nM, *k*_off_ of 7.7 × 10^−4^ s^−1^; Fig. 1D and Supplementary Table S2), making it a promising candidate as a fiducial marker in SPA cryo-EM of RNAs.

### Single particle cryo-EM analysis of U1A–RNA-Fab complex

To evaluate the potential of using the selected Fab1R as a universal fiducial marker for determining U1A–RNA structures by cryo-EM, we tested the cIL ribozyme with a grafted U1hpII loop [26]. To further enhance particle visibility in cryo-EM micrographs, the cIL–U1A–Fab1R complex was purified in the presence of a secondary Fab (sfFab18) bound to an engineered variant of *Streptococcus* Protein G (PGA1, [35, 37]). The sfFab18–PGA1 reagent was specifically designed to increase the overall mass and modify particle shape. By increasing size and reshaping the complex, sfFab18–PGA1 also helps reduce preferred orientation and supports broader angular sampling, leading to more reliable 3D reconstructions. A detailed description of the design and broader use of the sfFab18–PGA1 fiducial will be provided in a separate study.

**Figure 2.**
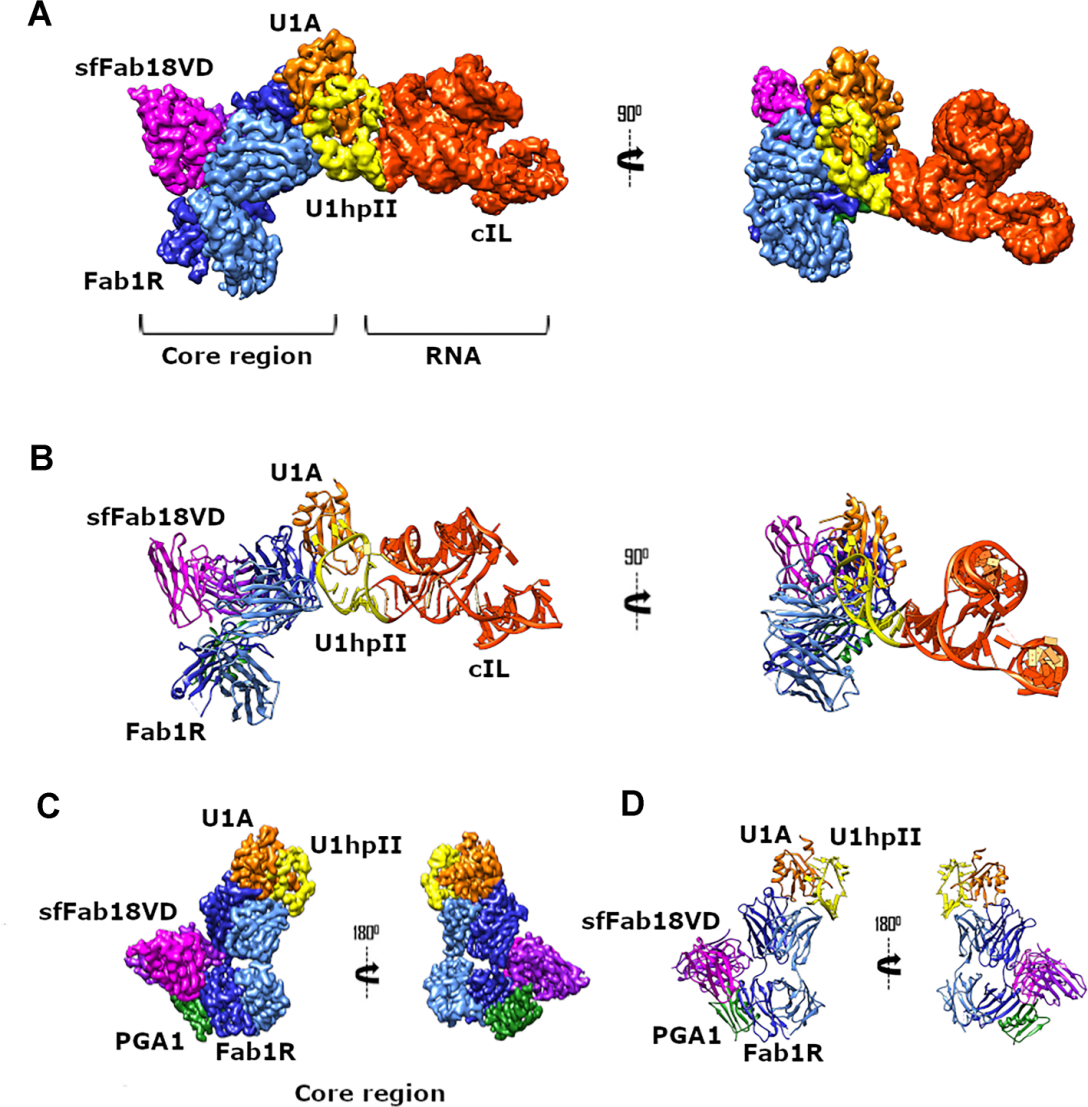
Overall cryo-EM structure of the cIL–U1A–Fab1R–PGA1–sfFab18 complex. (**A**) Composite cryo-EM map of the entire complex shown in two orientations. (**B**) Two orientations of the atomic model of the full complex, depicting both the core and RNA components. (**C**) Cryo-EM focused density map of the core structure shown in two orientations, with corresponding atomic model views (**D**). Color scheme used in the models: cIL (red), U1A (orange), U1hpII loop (yellow), Fab1R heavy chain (blue), Fab1R light chain (light blue), PGA1 (green), VD of sfFab18 heavy chain (pink), and VD of sfFab18 light chain (purple).

For the cIL–U1A–Fab1R–PGA1–sfFab18 quaternary complex, we collected 10 696 movies, resulting in a 3D reconstruction with a global resolution of 3.04 Å (Supplementary Fig. S5C and Supplementary Table S3). The complex consists of a stable core comprising U1A, the U1hpII RNA loop, Fab1R, PGA1, and sfFab18, along with a flexible RNA region (Fig. 2A and B). During refinement, the constant domains of sfFab18 were omitted due to their flexibility and the lower resolution of the corresponding map regions. Focused refinement of the core complex in *cryoSPARC* [38], which involved particle subtraction followed by local refinement, improved the resolution of this region to 2.9 Å (Fig. 2C and D, Supplementary Fig. S5D and E). Similarly, *Relion* [40] 3D classification combined with particle subtraction and *cryoSPARC* refinements using an RNA-specific mask yielded an improved cIL map, albeit at a lower global resolution of 4.32 Å (Supplementary Fig. S5D and F). The composite cryo-EM density map of the core and RNA regions is shown in Fig. 2A. The resulting map and structural model reveal that Fab1R binds specifically at the interface between the U1A protein and the U1hpII binding loop of the adjacent cIL molecule. This high level of specificity indicates that the selected Fab could serve as a universal fiducial for various RNA–U1A complexes.

### U1A–RNA–Fab interface

The focused refinement of the core region, including U1A:U1hpII, Fab1R, PGA1, and the variable domains of sfFab18, has improved the map quality of the complex, achieving a local resolution range of 2.3 to 3.9 Å (Supplementary Fig. S5E). The resulting map clearly shows a well-defined interface between Fab1R and the U1A:U1hpII loop of the RNA (Fig. 2C and D, Supplementary Figs S5E and S6). This high-quality density facilitated the construction of an initial structural model based on the crystal structures of U1A bound to the U1hpII loop and Fab. The Fab1R CDRs were manually modeled in *Coot* [42], followed by subsequent optimization and real-space refinement using *phenix.real_space_refine* [43]. U1A maintains the same interactions with the U1hpII loop as observed in the crystal structure, and the U1A:U1hpII conformation within the cryo-EM complex aligns closely with the previously reported structure [26].

**Figure 3.**
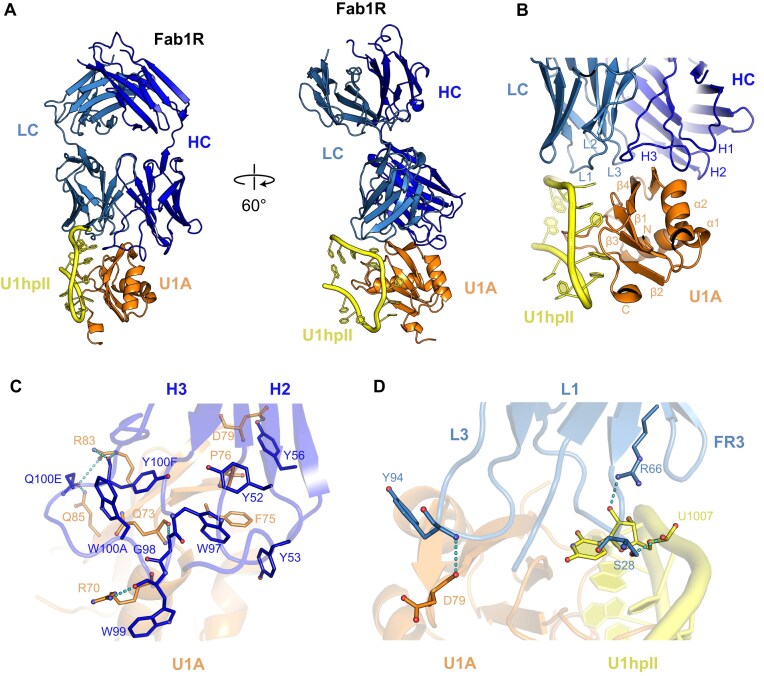
Molecular basis of Fab1R recognition of the U1hpII:U1A complex. (**A**) Cryo-EM structure of the U1hpII:U1A–Fab1R ternary complex. (**B**) Overview of the U1hpII:U1A–Fab1R interface, with the CDRs and the secondary structure elements of U1A indicated. Fab1R binds a composite epitope formed by both U1A and U1hpII. (**C** and **D**) Detailed interactions mediated by the heavy and light chains of Fab1R, respectively. The heavy chain utilizes CDRs H2 and H3 to form a network of hydrogen-bonding and hydrophobic interactions with U1A, whereas the light chain CDRs L1 and L3 forms hydrogen bonds with U1hpII and U1A, respectively. Cyan dashes indicate heteroatoms within hydrogen-bonding distance (3.6 Å). Residues and nucleotides are presented as cylinder models with oxygens in red and nitrogens in blue. Amino acid numbering for Fab1R follows the Kabat convention [50].

At the interface, residues from the Fab heavy chain interact with the U1A protein, while residues from the light chain contact both the U1A protein and the U1hpII loop region (Fig. 3A and B, Supplementary Fig. S8). According to PDBePISA analysis (www.ebi.ac.uk/pdbe/pisa), the total buried surface area of the U1A:U1hpII–Fab1R complex is 1048 Å². Of this, 826 Å² corresponds specifically to the U1A–Fab1R interface, with the heavy chain providing most of the contact surface (580 Å²) and the light chain contributing the remaining 246 Å². Fab binding to U1A is facilitated by a network of hydrophobic, electrostatic, and hydrogen-bonding interactions. U1A residues located on helix α2, strand β4, and the loops connecting β1–α1, α1–β2, and α2–β4 primarily interact with the CDRs H2 and H3 of the Fab heavy chain and L1 and L3 of the light chain (Fig. 3B and Supplementary Fig. S8). PDBePISA predicted a total of 10 hydrogen bonds: seven between U1A and the heavy chain CDR residues, and three with light chain CDR and framework residues. Key interacting residues include Y56, G98, W99, Q100E, and Y100F from the H2 and H3 regions of the heavy chain; S28 and Y94 from the L1 and L3 regions, as well as R66 from the light chain. This bonding network involves U1A residues R70 (α2), Q73 (α2–β4), D79 (α2–β4), R83 (β4), and Q85 (β4) (Fig. 3C and D). Hydrophobic interactions are mainly mediated by multiple tyrosine and tryptophan residues on the CDRs, specifically Y52, Y53, Y56, W97, W99, W100A, and Y100F in H2 and H3, as well as Y94 in L3.

Conversely, the U1hpII-binding loop primarily interacts with residues of the Fab light chain, with an interface area of 222 Å² as calculated by PDBePISA. Fab binding to the U1hpII loop is driven by electrostatic and hydrogen bonding interactions (Fig. 3D and Supplementary Fig. S8), forming two hydrogen bonds between light chain residues S28 and R66 and nucleotide U1007 of the U1hpII loop. Additionally, lysines K20 and K22 of U1A form hydrogen bonds with the phosphate groups of RNA nucleotides A1001 and C1003 adjacent to the U1hpII loop in the complexed structure (Supplementary Fig. S8B).

Notably, the Fab recognizes a composite epitope formed by amino acid residues of U1A together with nucleotides from the U1hpII RNA motif. The absence of Fab binding to U1A alone indicates that, in the absence of RNA, certain regions of U1A are too flexible or disordered to facilitate Fab interaction. RNA binding likely stabilizes the local structure of U1A, creating a defined surface necessary for Fab recognition. It is well established that conformational rearrangements of the C-terminal helix and the β2–β3 loop of U1A (Fig. 3B) are crucial for RNA recognition [51–53]. Without RNA, the C-terminal helix of U1A adopts multiple conformations [54], which may further impair Fab binding.

### Cryo-EM structure of cIL ribozyme

The catalytic activity7 of cIL ribozyme depends on proper folding into a 3D structure and the presence of Mg²⁺ ions. Since cIL can extend a primer-template pair by adding nucleotides, it serves as a useful model for designing more complex RNA polymerase ribozymes [55–57]. The full-length cIL ribozyme, with a grafted U1hpII loop, is a 44.1 kDa RNA consisting of 137 nucleotides [26]. Its crystal structure exhibits a tripod-like shape formed by three domains, each containing seven helices and three junction regions: P1–P2, P3–P6–P7, and P4–P5, along with J1/2, J1/3, and J3/4. The inserted U1hpII loop is located at the stem of the P5 helix [26].

**Figure 4.**
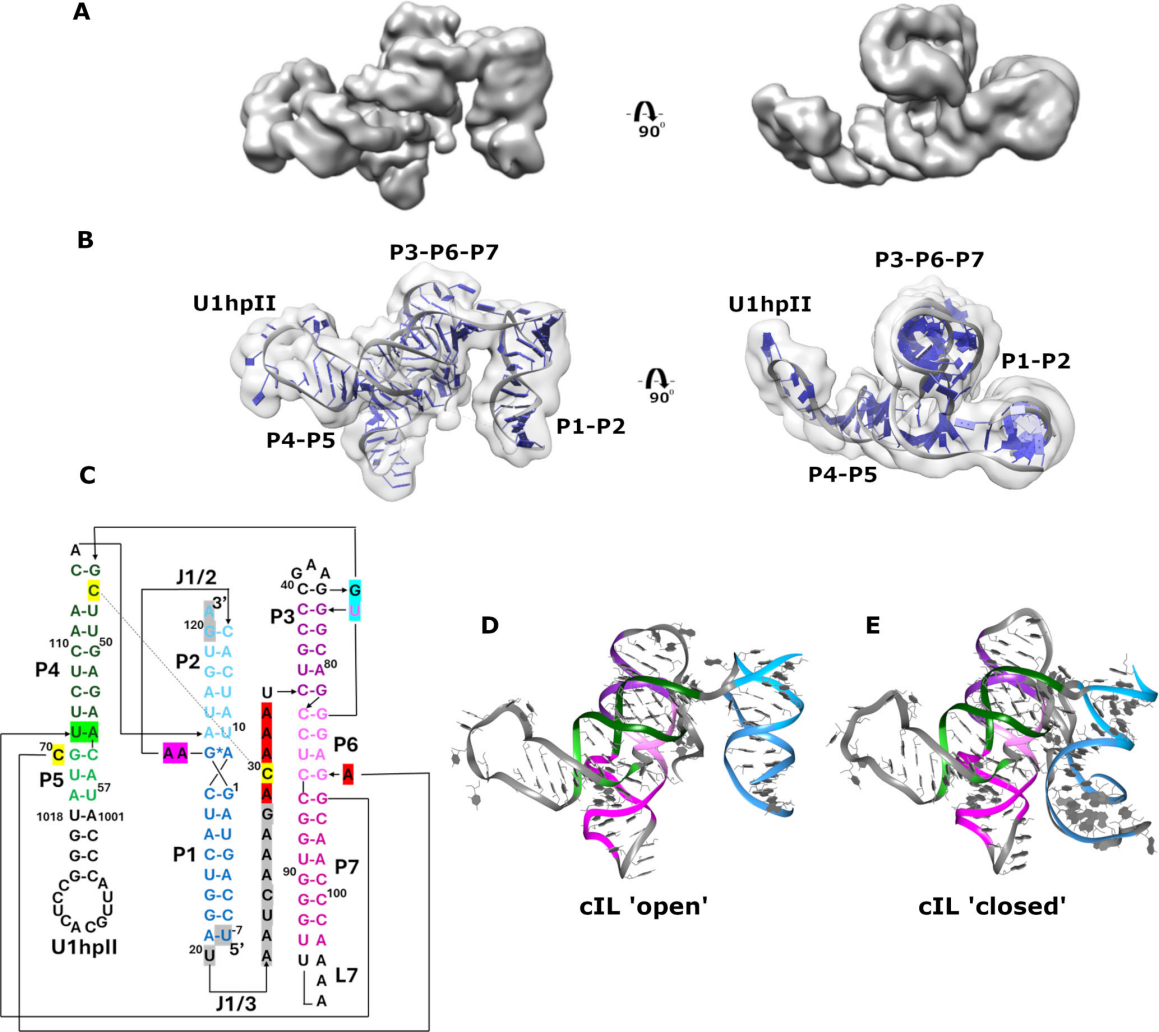
Cryo-EM 3D reconstruction of cIL RNA. (**A**) Cryo-EM density map of cIL shown from two different views. (**B**) Two views of the RNA structural model fitted into the cryo-EM density. (**C**) The secondary structure of cIL with each domain colored distinctly. The black arrow indicates the backbone direction. Nucleotides absent from the cryo-EM model are highlighted in gray. Nucleotides participating in key ternary interactions within the catalytic domain are highlighted in red (A3, A32, A33, A29, A71), yellow (C30, C47, C70), cyan (G45, U76), and green (U106, A54), while those in the J1/2 linker are highlighted in pink. The numbering scheme for cIL was adopted from the crystal structure [26]. In this construct, loop L5 and the two closing base pairs of the ribozyme (nucleotides 58–66) were replaced with a larger loop and four closing base pairs corresponding to the U1hpII hairpin (nucleotides 1001–1018). (**D**) Ribbon representation of the cryo-EM structure of cIL in the “open” complex with colored domains. (**E**) Ribbon representation of the crystal structure of cIL in the “closed” complex with colored domains.

In the global cryo-EM map of the cIL–U1A–Fab1R–PGA1–sfFab18 quaternary complex, the cIL region was poorly resolved (Supplementary Fig. S5C). To improve this, we performed focused 3D classification and local refinement, which enabled separate refinement of the RNA and recovered density in the disordered region. The resulting cIL reconstruction achieved a local resolution of 3–7.5 Å, reflecting significant flexibility in certain ribozyme domains (Supplementary Fig. S5F). The 3D reconstruction showed that cIL maintains the overall three-domain fold (Fig. 4A); however, fitting the cIL crystal structure into the map revealed notable conformational differences. To build the cIL model into the electron density map, we used *auto-DRRAFTER* [5], followed by RNA structure optimization in *Phenix* [41] and *ERRASER* [44]. The resulting structural model indicates that cIL preserves its global fold (Fig. 4B). Notably, the first domain (P1–P2) is shifted upward and rotated relative to the central helical axis, while the ribozyme continues to maintain its secondary structural elements (Fig. 4C and D). The density map and top-scoring cIL models generated by *auto-DRRAFTER* suggest that the joining region J1/3 is more flexible, leading to low resolvability (Supplementary Fig. S9C). As a result, we have omitted the disordered portion of the J1/3 region (nucleotides 20–28) from our final structure.

Comparison of the X-ray and cryo-EM structures reveals that the cryo-EM model of cIL likely depicts an “open” complex, where the substrate helix P1 is undocked from the catalytic domains (P4–P5 and P3–P6–P7) (Fig. 4D and E). In this structure, J1/2, the GAA linker connecting P1 and P2, appears tucked into the major groove of P2, which may help stabilize the open conformation (Supplementary Fig. S10A). Conversely, the crystal structure shows a “closed” complex, in which the two adenines of the J1/2 linker interact with P4 CG, bringing the P1-P2 domain closer to the catalytic core (Fig. 4E). Furthermore, in the crystal structure, the adenosine triplet A25–A26–A27 of J1/3 is docked into the minor grooves of P1 and P6 and contacts P1 near its fourth and fifth base pairs. These interactions support P1 through stacking and hydrogen bonds at the primer-template junction, resulting in a conformation consistent with a catalytically active state [26]. In the cryo-EM structure, density for A25–A26–A27 is poorly resolved, indicating greater flexibility and allowing P1 to assume alternative conformations. The ability of A25–A26–A27 to switch between ordered, engaged positions and disordered, disengaged positions suggests that this triplet likely has a role in conformational changes between docked and undocked states of the ribozyme.

Despite these differences, comparison of the catalytic domains (P4–P5 and P3–P6–P7) shows good overall agreement between the X-ray and cryo-EM structures, with key tertiary interactions maintained (Fig. 4C and Supplementary Fig. S10B–F). These include the A-minor triad (A31–A32–A33) interactions with P6, stacking between A29 and A71, stacking between C30 and C47, stacking between G45 and U76, the unpaired C70, and the U106–A54 base pair [9, 26]. Therefore, our cryo-EM structure of cIL supports a model in which the ribozyme’s catalytic domains can fold and form native tertiary interactions independently of substrate docking. Consistent with this, the recently reported cryo-EM structure of the dimeric triplet polymerase ribozyme (TPR) apoenzyme, which includes the catalytically active 5TU and the inactive t1 subunit, also maintains a cIL-like catalytic core with similar tertiary interactions [58]. Notably, the authors attempted to capture the TPR holoenzyme in a substrate-bound state by adding the substrate helix in trans; however, 3D reconstruction revealed only the apoenzyme, highlighting the transient nature of substrate docking.

Although the cryo-EM structure was acquired in the presence of 5 mM Mg²⁺, which is sufficient to support ribozyme folding [59], the effective Mg²⁺ concentration might be lower because of the high RNA concentration in the cryo-EM sample and the number of metal ions needed for folding. As a result, our structure likely captures cIL under conditions that promote a lower effective Mg²⁺ level. The observed “open” state may therefore reflect magnesium-dependent RNA folding: while Mg²⁺ stabilizes the compact, “closed” conformation by promoting tertiary interactions and substrate docking, lower effective Mg²⁺ levels shift the equilibrium toward more extended, “open” states. This dynamic balance likely represents an important functional feature of cIL, allowing the flexibility needed for substrate docking and catalysis.

## Discussion

Determining RNA structures by cryo-EM remains a major challenge because RNA molecules are naturally flexible, often small, and can adopt multiple dynamic conformational states. These characteristics lead to pronounced structural heterogeneity, hinder accurate particle picking, alignment, and ultimately limit the achievable resolution. Consequently, reliably building models of RNAs is much more challenging than building models of larger, more rigid macromolecular complexes. These difficulties require innovative strategies to improve the visibility and structural understanding of RNA molecules, such as engineered self-assembled RNA homomeric structures or scaffolding methods [1–3]. Although these techniques have enabled high-resolution structures of RNAs using cryo-EM, they may also sometimes cause artificial effects on RNA dynamics and biologically relevant conformations.

An alternative and promising strategy employs antigen-binding fragments (Fabs) as fiducial markers. While RNA scaffolding approaches, such as group II intron fusions [3], can also increase molecular mass and aid particle alignment, they do so by embedding the RNA within a larger structural framework. In contrast, Fabs bind directly to the target RNA, increasing its effective molecular mass and introducing high-contrast features that enhance particle picking and alignment in SPA cryo-EM, while being unlikely to alter the native RNA conformation. This makes Fab-assisted visualization a promising alternative that preserves RNA flexibility while addressing the alignment challenges of dynamic RNA molecules. However, the traditional method of selecting Fabs for specific RNA targets is slow and complicated by the structural diversity and flexibility of RNA, as well as the limited availability of phage-display libraries for RNA epitopes. To address these issues, this study introduces a universal Fab that targets a conserved U1A–RNA epitope, specifically the U1hpII loop. The U1hpII (U1 hairpin II) loop is a short, well-characterized RNA stem-loop motif derived from U1 small nuclear ribonucleoprotein (U1 snRNP). It forms a defined hairpin structure that is specifically recognized with high affinity and sequence selectivity by the U1A protein [47, 48, 51].

Over the past three decades, the U1A–U1hpII interaction has become a well-established and canonical example of sequence-specific RNA–protein recognition. Structural and biochemical studies have shown that U1A binds the U1hpII loop primarily through specific contacts with the loop nucleotides and one or two closing stem base pairs, while the remainder of the stem serves as a stable structural scaffold [47, 48]. Notably, the U1hpII loop can be engineered into diverse RNA molecules without perturbing their native folds or functional activity, making it a versatile tool in RNA structural biology. In addition to its incorporation into the RNA self-splicing group I intron [60], K-turn RNA [11], SAM-VI riboswitch [61], NAD^+^-I riboswitch [62], L-glutamine riboswitch [63], glycine riboswitch [64], c-di-AMP riboswitch *ydaO* [65], c-di-GMP riboswitch [66, 67], ThiM riboswitch [68], tetracycline adapter [69], the U1hpII element has also been appended to other structured RNAs, such as the HDV ribozyme [8, 70], the aminoacyl tRNA synthetase ribozyme [71], cIL ribozyme [9, 26], and *glmS* ribozyme [72], to provide well-defined protein docking sites. The U1hpII has further been employed as an RNA affinity tag on single-stranded RNAs, including small non-coding RNAs (snoRNAs), where it provides a discrete protein-binding site for U1A and enables efficient isolation of RNA-protein complexes [73]. Taken together, the extensive structural and biochemical characterization of U1hpII and the well-defined U1A-U1hpII complex establishes U1 hairpin as an ideal RNA recognition element, selected to serve as a robust and reliable Fab-binding component in the present study.

Our findings illustrate that grafting the U1hpII loop onto target RNAs facilitates the formation of a stable U1A–RNA complex that can be recognized by the selected Fab1R with high specificity and affinity. SPR and ELISA assays have confirmed that Fab1R binds the U1A:U1hpII complex with nanomolar affinity and slow dissociation rates, properties that are essential for effective cryo-EM applications. Notably, Fab1R maintained its binding properties across diverse RNA systems, including Spinach RNA and the cIL ribozyme [14, 16, 26], underscoring the versatility and broad applicability of the U1A–RNA–Fab module.

The cryo-EM structure of the class I ligase ribozyme (cIL RNA), which includes the grafted U1hpII loop and is in complex with U1A, Fab1R, and PGA1–sfFab18, was determined at a global resolution of 3.04 Å. Focused refinement further improved map quality, achieving a local resolution of approximately 2.3 Å at the Fab-binding interface, enabling detailed visualization of key interactions. The structure of the quaternary complex showed that Fab1R binds at the interface between the U1A protein and the U1hpII loop, engaging both protein and RNA through hydrophobic, electrostatic, and hydrogen-bonding interactions. The interface area, as calculated by PDBePISA, was large, with the heavy chain of the Fab contributing most contacts with U1A, and the light chain interacting with both U1A and the U1hpII loop. Surprisingly, Fab1R does not form extensive interactions with the base pairs that connect the hairpin loop to the ribozyme, nor does it extend contacts into the ribozyme core. The four base pairs linking the hairpin to the ribozyme adopt canonical A-form geometry and show no detectable distortion. Together, these observations indicate that Fab binding primarily stabilizes the U1A-hairpin module itself rather than constraining the adjacent ribozyme structure. Structural superposition of the U1hpII–U1A–Fab1R–PGA–sfFab18 complex with 20 RNA structures solved using the U1hpII–U1A crystallization module further demonstrates that the complex would not be expected to introduce steric clashes with any of these previously characterized RNAs (Supplementary Fig. S11). This dual recognition mode confers both high specificity and stability to the Fab–U1A–RNA complex without perturbing RNA conformation, supporting its utility as a universal fiducial marker for cryo-EM studies.

Additionally, we incorporated sfFab18–PGA1, a novel engineered fiducial designed to overcome intrinsic limitations in cryo-EM analysis of small antigens, as an auxiliary component to facilitate structural studies. Our unpublished results show that adding the sfFab18–PGA1 module, which contributes ∼60 kDa to the overall molecular mass, enhances particle geometry without affecting the biochemical properties of the primary Fab–antigen complex. These changes promote a broader angular distribution, resulting in improved-quality reconstructions. Although the direct impact of sfFab18–PGA1 on RNA map quality was not explicitly assessed in this study, we anticipate that sfFab18–PGA1 provides effective complementary support to Fab1R, enhancing its utility for RNA structure determination by SPA cryo-EM. Importantly, the combined Fab1R–PGA1–sfFab18 module has the potential to address challenges associated with imaging small RNAs and RNA–protein assemblies.

The 3D reconstruction of cIL at 4.32 Å resolution demonstrated that Fab binding to the U1A:U1hpII loop, located at the base of helix P5 in the catalytic domain, maintains the native RNA fold, demonstrating that Fab association is structurally compatible with the ribozyme. Notably, the cryo-EM structure likely captured cIL in an “open” conformation, with the substrate helix undocked from the catalytic domains. In contrast, previously reported crystal structures depict cIL in a “closed” conformation corresponding to the catalytically active state [8, 16, 25]. The observation of the “open” state suggests that the catalytic domains of cIL can fold and interact naturally, independent of substrate docking. The ability to adopt undocked conformations may be a key mechanism for translocation during polymerization. Together, these findings indicate that the modular U1A–RNA–Fab system maintains the native architecture of the RNA while preserving its inherent conformational flexibility—an essential feature for the biological function of catalytic RNAs. Although the cryo-EM data were collected at reduced Mg²⁺ concentrations, this likely supported the dynamic behavior of the RNA. In contrast, higher Mg²⁺ concentrations would stabilize a more “closed” RNA conformation and could improve the resolution of the cryo-EM map.

Fab binding improves particle visualization and alignment, yet it does not restrict RNA conformational dynamics. As a result, flexible regions can limit local resolution, as observed for the cIL ribozyme in this study. Overall map quality, therefore, depends largely on the intrinsic conformational homogeneity of the RNA construct. Achieving optimal results requires careful optimization of buffer composition, divalent ion concentration, and RNA architecture to balance proper folding with sample stability. Within this framework, the U1A–RNA–Fab platform serves as a practical, “off-the-shelf” antibody-based tool to expand the range of RNAs amenable to SPA cryo-EM but still relies on these standard optimizations to maintain RNA homogeneity and particle stability during data collection.

The successful implementation of the U1A–RNA–Fab module in the cIL ribozyme highlights the potential of this approach in RNA structural biology. By enabling the use of a single Fab as a universal fiducial marker, either alone or in combination with secondary fiducial sfFab18–PGA1, this methodology streamlines Fab selection and accelerates the structural analysis of various RNAs. The ability to engineer the U1hpII loop into different RNA molecules, thereby enabling high-affinity binding to Fab1R, opens new avenues for studying the conformational dynamics and functional mechanisms of biologically and medically important RNAs. Moreover, the cryo-EM workflow developed here can be applied to other RNAs containing the U1hpII loop, providing a general strategy for map reconstruction and initial model building.

In summary, this study establishes a robust and generalizable method for improving cryo-EM structure determination of RNAs by targeting a conserved protein–RNA epitope with a universal Fab. The method is expected to enable high-resolution structural analysis of diverse RNAs, thereby enhancing our understanding of RNA biology and opening new avenues for RNA-based therapeutics and research.

## Supplementary Material

gkag502_Supplemental_File

## Data Availability

The plasmids of the synthetic antibodies characterized in this work will be made available from the corresponding author upon reasonable request. The coordinates have been deposited in the Protein Data Bank (PDB) with accession codes PDB ID: 9YXV (structure of the core region) and 9Z6I (structure of the RNA region). The cryo-EM maps have been deposited in the Electron Microscopy Data Bank (EMDB) under accession codes EMD-73618 (focused map of the core region), EMD-73840 (focused map of the RNA region), EMD-73655 (global map of the complex), and EMD-75812 (composite map of the complex).
