# Supplementary material for: A universal Fab targeting a conserved U1A–RNA epitope for RNA structure determination by cryo-EM
Source: Nucleic Acids Res. 2026 May 20;54(10):gkag502. doi: 10.1093/nar/gkag502 (PMC13187837; doi:10.1093/nar/gkag502)
Supplement: gkag502_Supplemental_File [file gkag502_supplemental_file.pdf]

Supplementary Table S1. Kinetic parameters of Fab1R binding to the U1hpII:U1A complex in distinct RNA contexts.

**Complex**	**K_D (nM)**	**k_{on} (× 10^5 M^{-1} s^{-1})**	**k_{off} (× 10^{-4} s^{-1})**
U1hpII:U1A	0.17	29	4.9
Spinach-U1hpII:U1A	0.68	19	13
cIL-U1hpII:U1A	2.3	0.79	18

Supplementary Table S2. Summary of CDR sequences and kinetic binding parameters for the Fabs identified in this study. Only the CDRs that were diversified in library E are included.

**Fab**	**L3**	**H1**	**H2**	**H3**	**K_D (nM)**	**k_{on} (× 10^5 M^{-1} s^{-1})**	**k_{off} (× 10^{-4} $M^{-1}s^{-1}$)**
1R	SSFYPI	IYSSSI	SIYPYSGYTS	SPWGWFWQYGQYSVAL	4.0 (4.5)*	1.6 (1.7)	6.6 (7.7)*
1-B8	STWQPI	VSSSSI	SIYPYSGYTS	WRRHGGWYQYHWAF	2.0	4.4	8.8
2R-D5	SSWYPI	VYSYSI	SIYSSYGYTY	MGYGAHFTGDGI	2.4	6.3	15
1-C5	STSYPI	IYSSSI	SIYPSYGYTS	FSQYKWYYWSDFSAF	3.1	10	31
1R-B1	FTSYPI	ISYSSI	SIYSSYGYTS	NYYYFADQISGEGL	3.1	16	48
1-A11	STSYPF	VSYSSI	SIYPSYGYTS	HTSWSYYWPIEGM	7.2	8.8	64
2R-B3	SSYMPI	FSSSSI	SIYSSYGYTS	LMYGYYFQYDAF	15	18	280
2R-A8	SSFYPV	VSSSSI	YIYPYSGYTS	QHGYQYLNYWYYQYDGL	45	7.3	330

Supplementary Table S3. Cryo-EM sample preparation, data collection, data processing, and model refinement statistics.

	**cIL-U1A-Fab1R-PGA1-sfFab18**	
Blotting volume, µL	3.5	
Concentration, µM	13	
Detergent, % (w/v)	0.1 (Octyl Maltoside)	
Grid type	300-mesh R 1.2/1.3 UltrAuFoil	
Blotting time, s	3	
Temperature, ^{0}C	10	
Humidity, %	100	
Microscope	Titan Krios	
Voltage, kV	300	
Magnification	81,000	
Spherical aberration	2.7	
Detector	Gatan K3	
Total exposure, e^-/Å^2	60	
Defocus range, µm	-0.9 - -2.5	
Pixel size, Å	1.068	
Software for data collection	EPU	
Total number of movies	10,696	
Symmetry imposed	C1	
Number of initial particles from 2D Classification	1,264,747	
Number of final particles	430,597	
Resolution full map (0.143 FSC, Å)	3.04	
Resolution of the core/RNA region (0.143 FSC, Å)	2.9/4.32	
B-factor for map sharpening, Å^2:		
Full map	-124.9	
Core region	-92.6	
RNA region	-184.5	
Map-to-model fit (Q score, %) for core/RNA region	0.54/0.24	
Model Refinement	Core Region	RNA Region
Clashscore, %	5.8	6.5
Protein geometry:		
Ramachandran outliers, %	0	
Ramachandran favored, %	96.75	
Cβ deviations >0.25Å	0	
Bad bonds, %	0	
Bad angles, %	0.02	
RNA geometry:		
Probability wrong sugar puckers, %	0	0
RNA backbone, %	0.32	0.59
Bad bonds (%)	0	0
Bad angles (%)	0	0

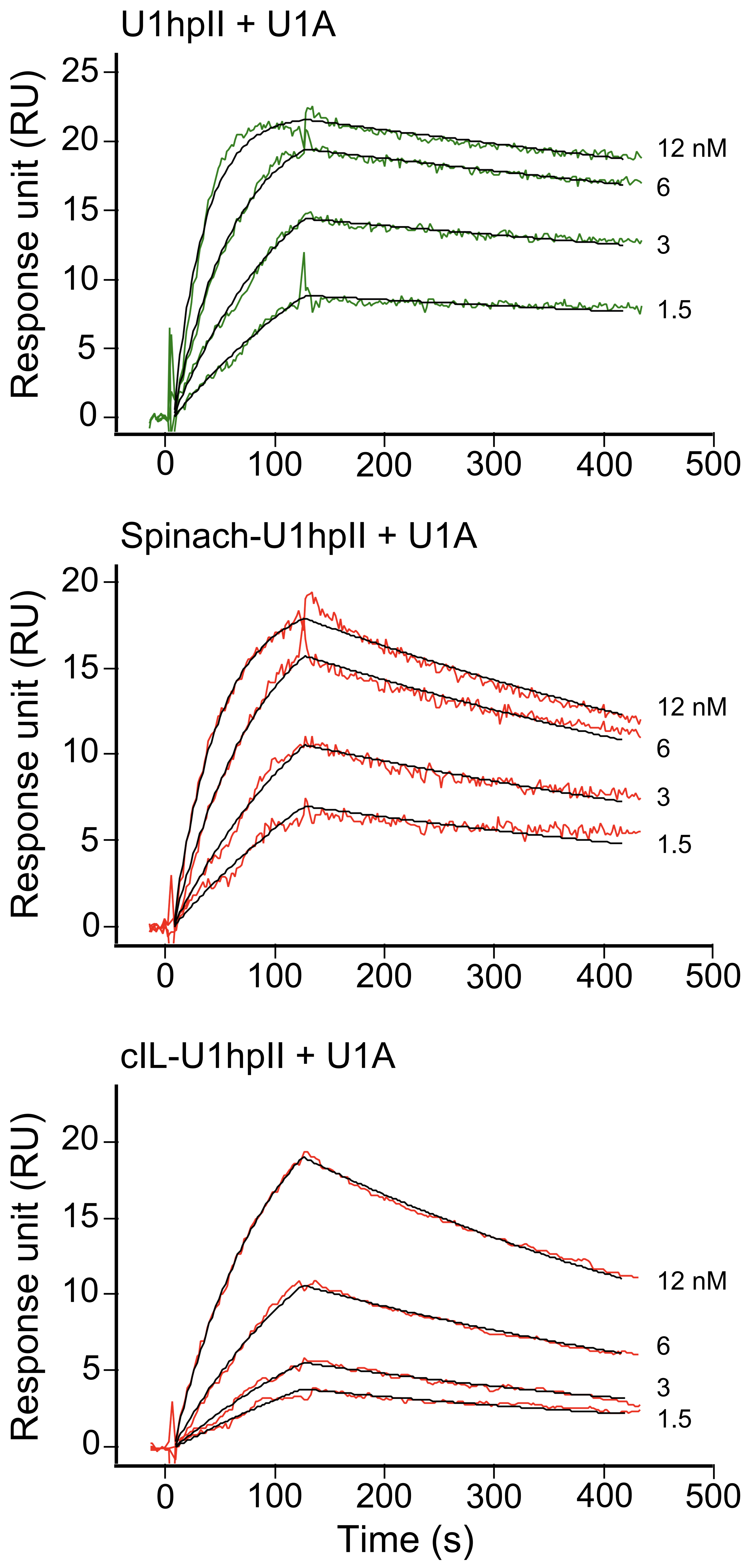

Supplementary Figure S1. Surface plasmon resonance sensorgrams showing kinetic analysis of U1A binding to the U1hpII stem-loop in the context of Spinach and cIL RNAs. The concentrations of analyte injected are indicated.

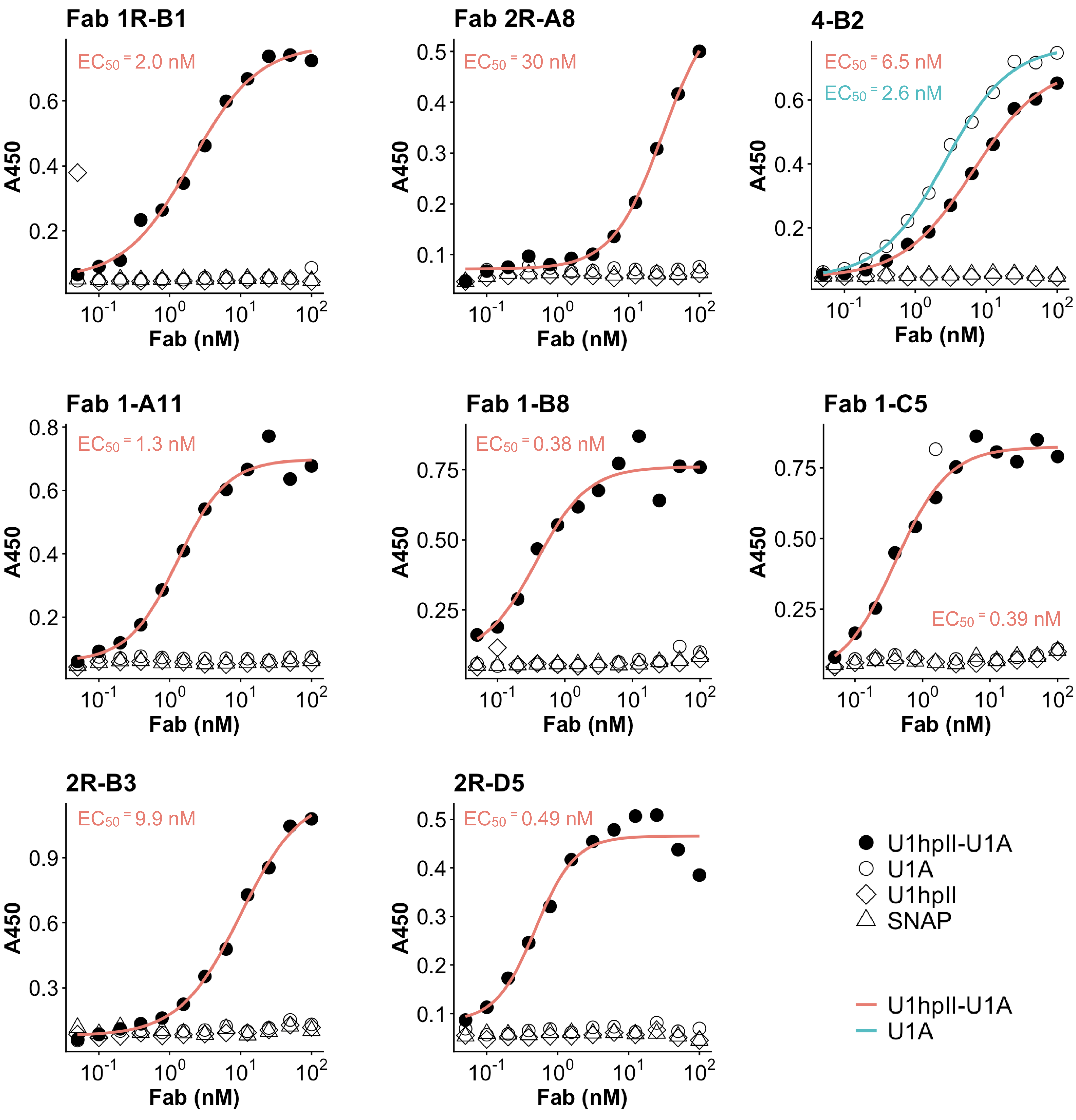

Supplementary Figure S2. ELISA binding curves demonstrating the EC_{50} and specificity other Fabs against U1hpII:U1A complex identified in the phage display selection in this study. The curves for samples with detectable binding are fitted with a non-linear sigmoidal function with variable slope for EC_{50} estimation. Binding responses are shown as absorbance at 450 nm plotted as a function of Fab concentration.

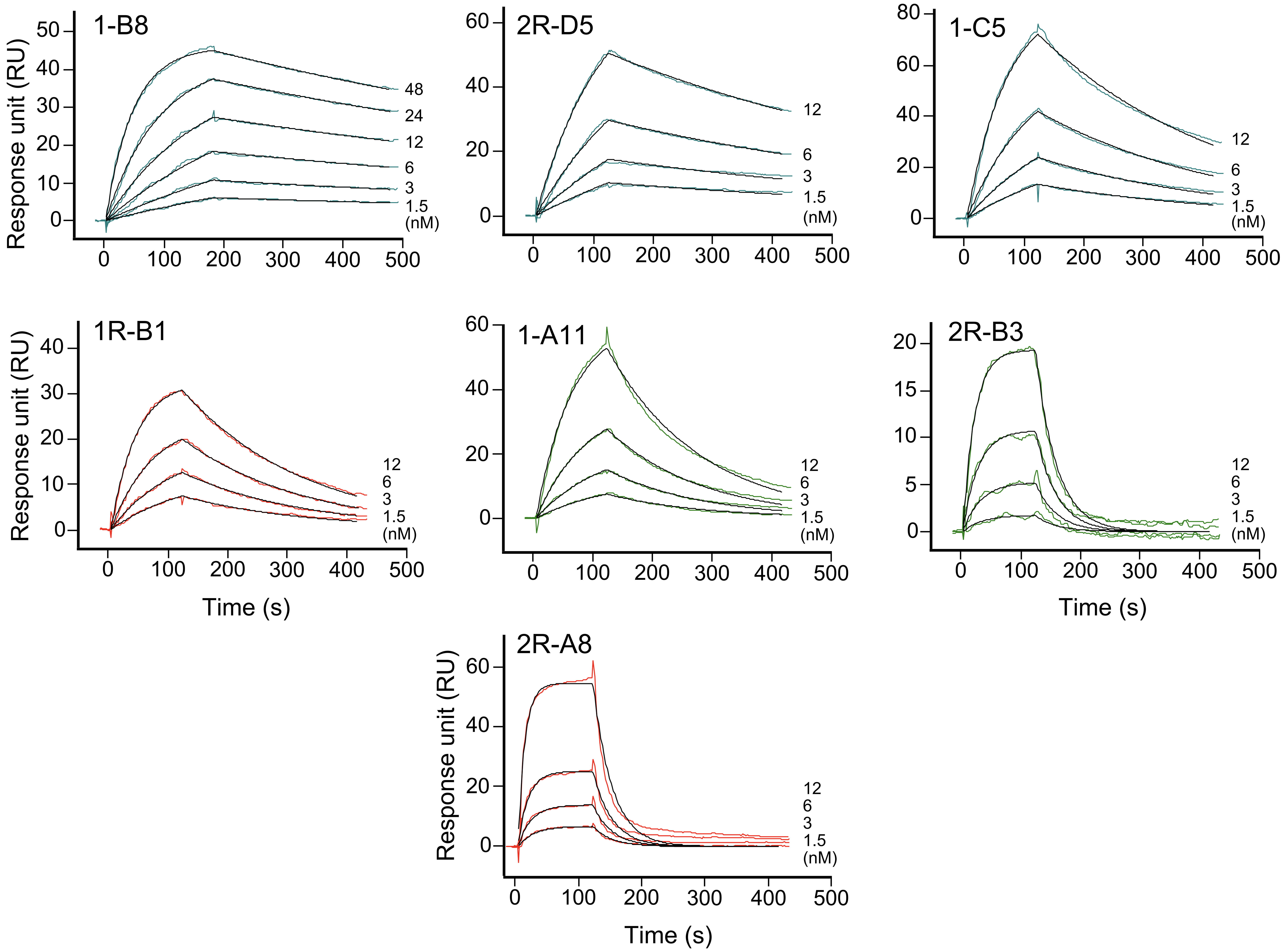

Supplementary Figure S3. Surface plasmon resonance sensorgrams showing kinetic analysis of U1hpII:U1A-specific Fabs binding to the U1hpII:U1A complex. The concentrations of injected analytes are indicated.

A

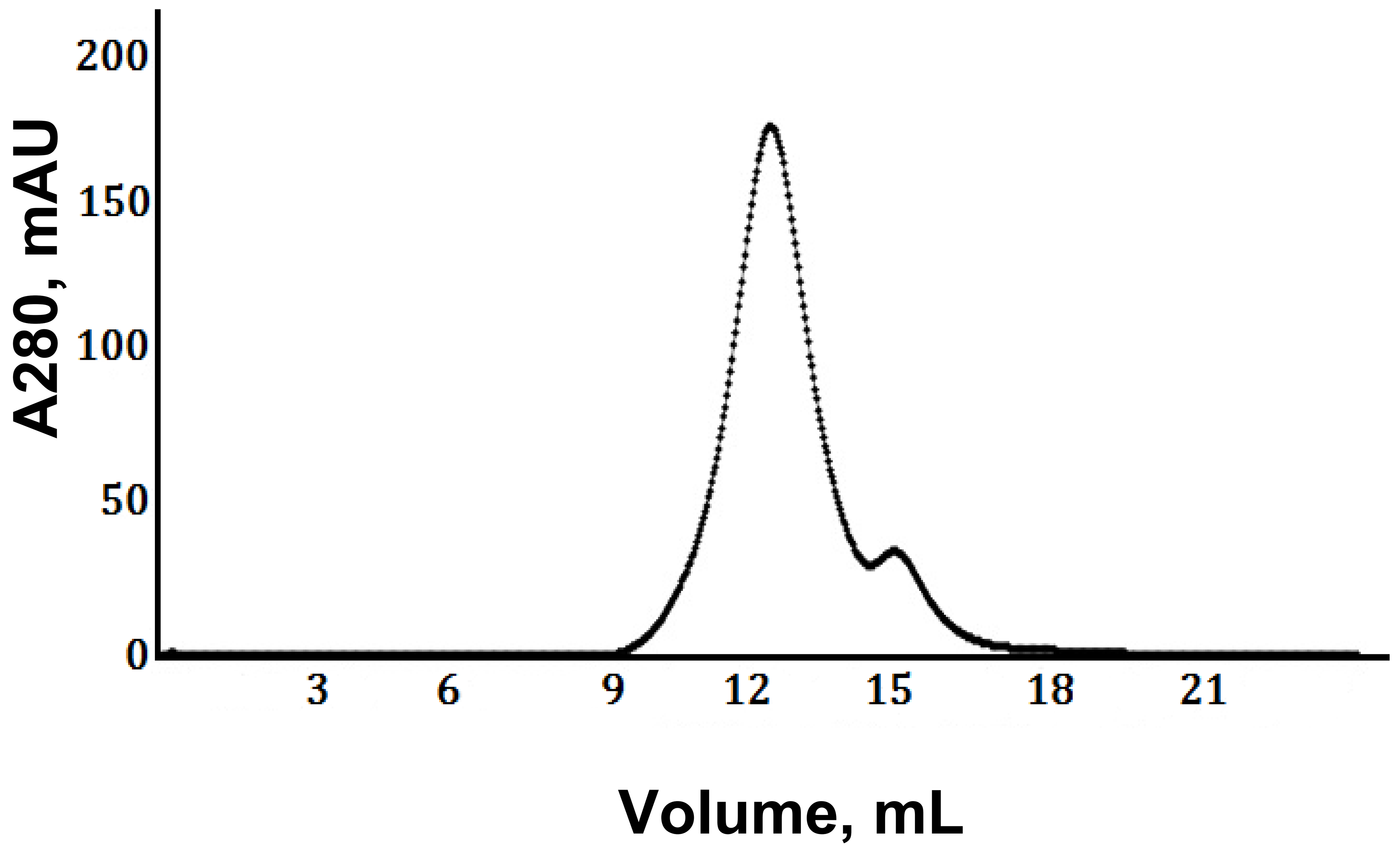

B

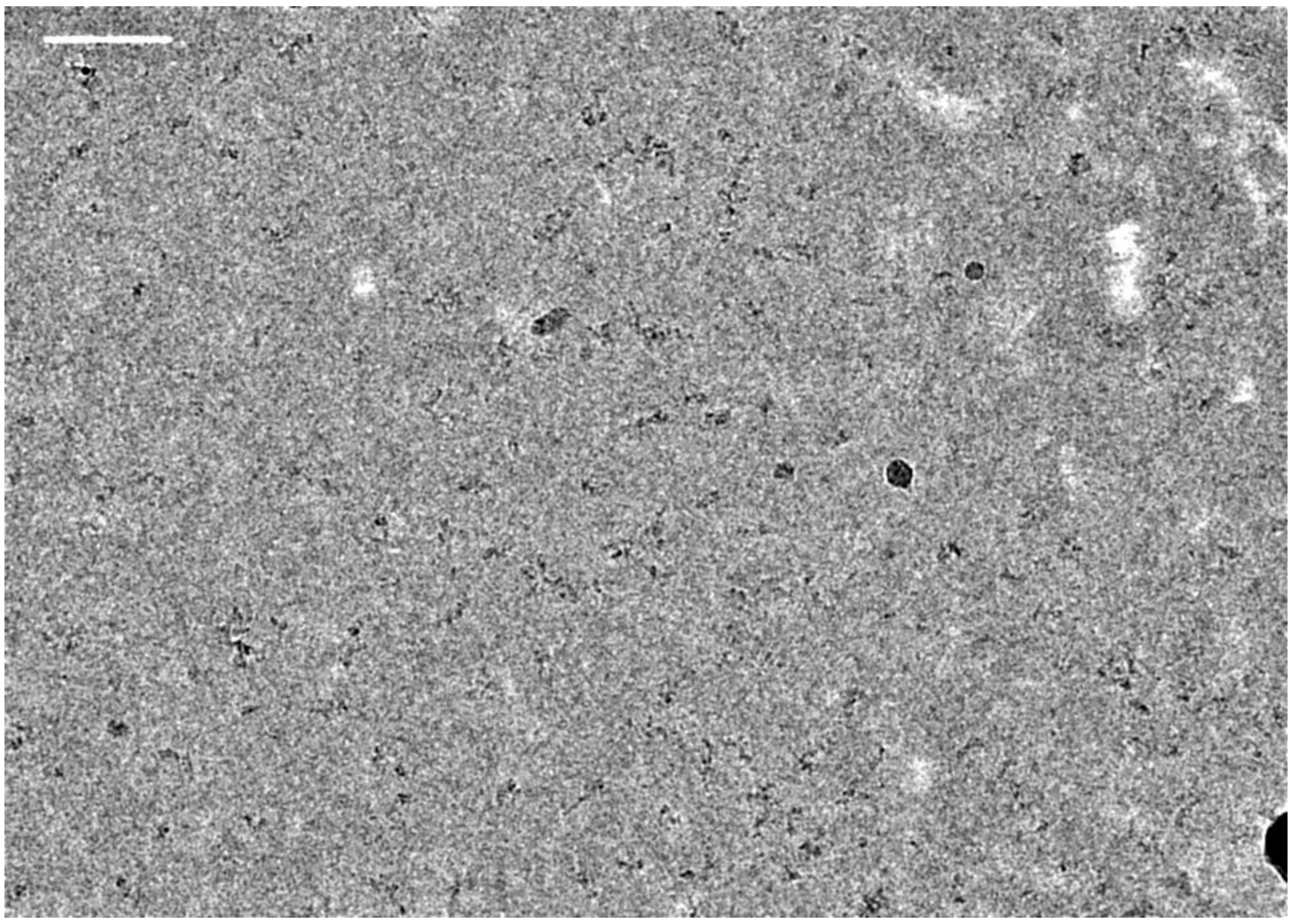

Supplementary Figure S4. (**A**) Size-exclusion chromatography elution profile of cIL-U1A-Fab1R-PGA1-sfFab18 complex. (**B**) A representative motion-corrected cryo-EM micrograph of the complex. The scale bar represents 100 nm.

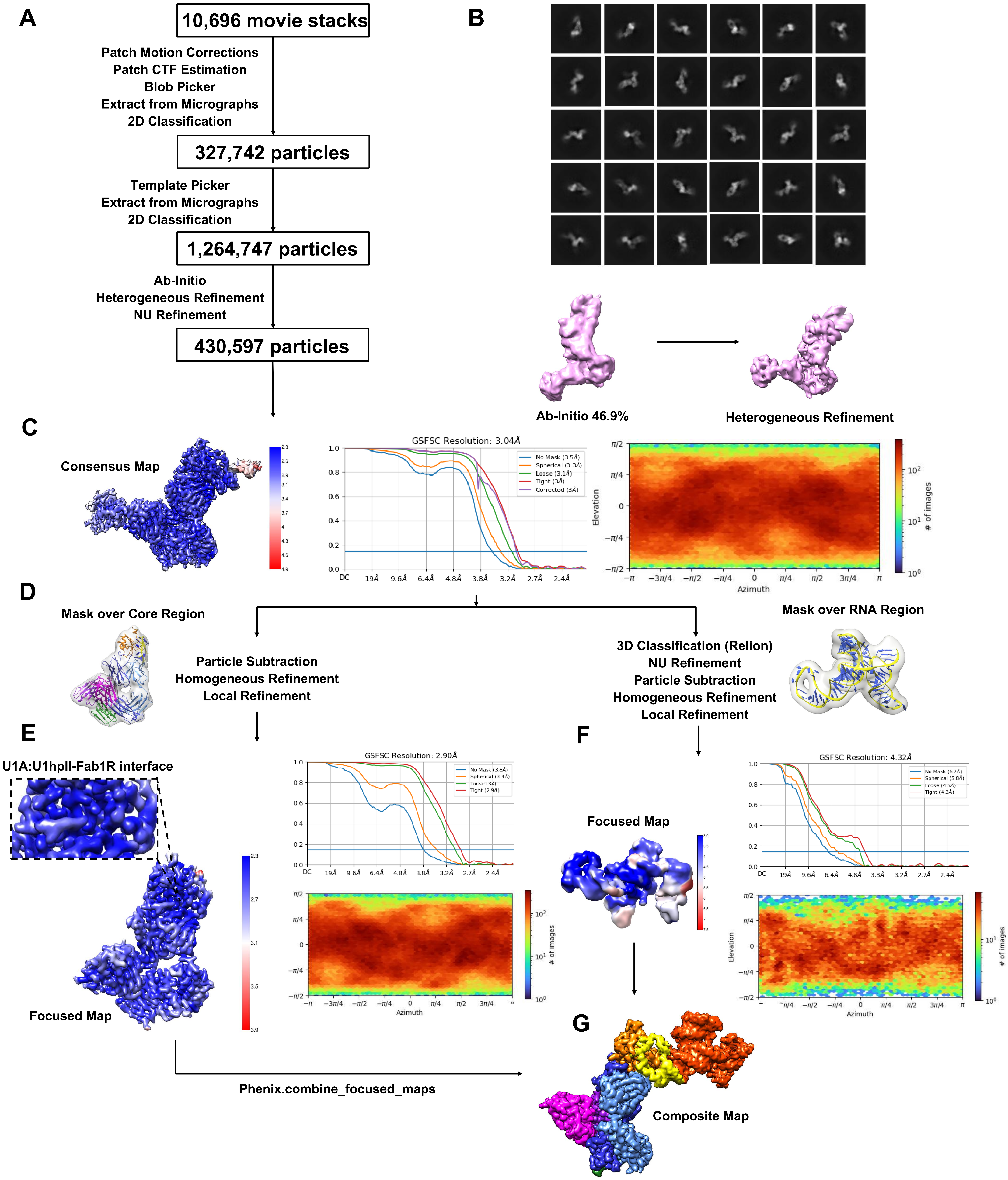

Supplementary Figure S5. (**A**) Cryo-EM 3D reconstruction workflow using *cryoSPARC*. (**B**) Representative 2D class averages alongside their corresponding low-resolution density maps obtained from *Ab-initio* and Heterogeneous refinements. (**C**) Cryo-EM consensus map of the complex, colored according to local resolution. (**D**) Masked volumes for local refinement, followed by the Cryo-EM focused map of the core region (**E**) or RNA region (**F**), colored according to local resolution. Close-up view of density at the U1A:U1hpII-Fab1R interface shown on inserted panel (**E**). (**G**) Composite map of the complex. The Gold-Standard Fourier Shell Correlation (GSFSC) plots were calculated by comparing the two independently reconstructed half-maps in *cryoSPARC*. The blue line indicates the 0.143 FSC threshold, corresponding to a nominal resolution. The angular distributions of particle projections was calculated in *cryoSPARC*, with the heat map representing the number of particles observed for each viewing angle.

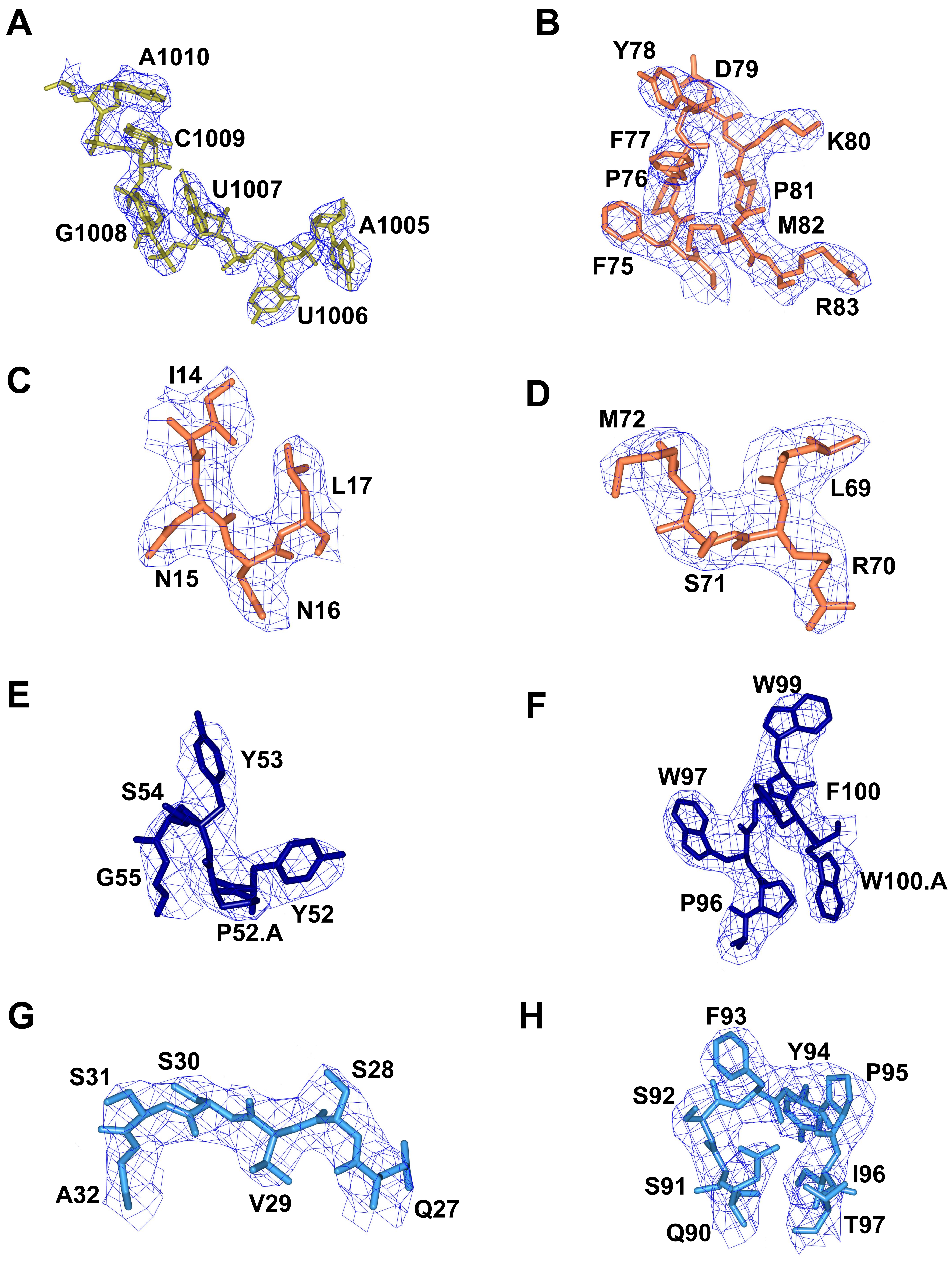

Supplementary Figure S6. Model-to-map fit of the U1A:U1hpII-Fab1R-PGA1-sfFab18 core complex. Residues at the U1A:U1hpII-Fab1R interface are shown as cylinders overlaid with the cryo-EM map (blue mesh). (**A**) U1hpII nucleotides. (**B**-**D**) U1A residues. (**E**, **F**) residues from the Fab1R CDRs H2 and H3. (**G**, **H**) residues from the Fab1R CDRs L1 and L3.

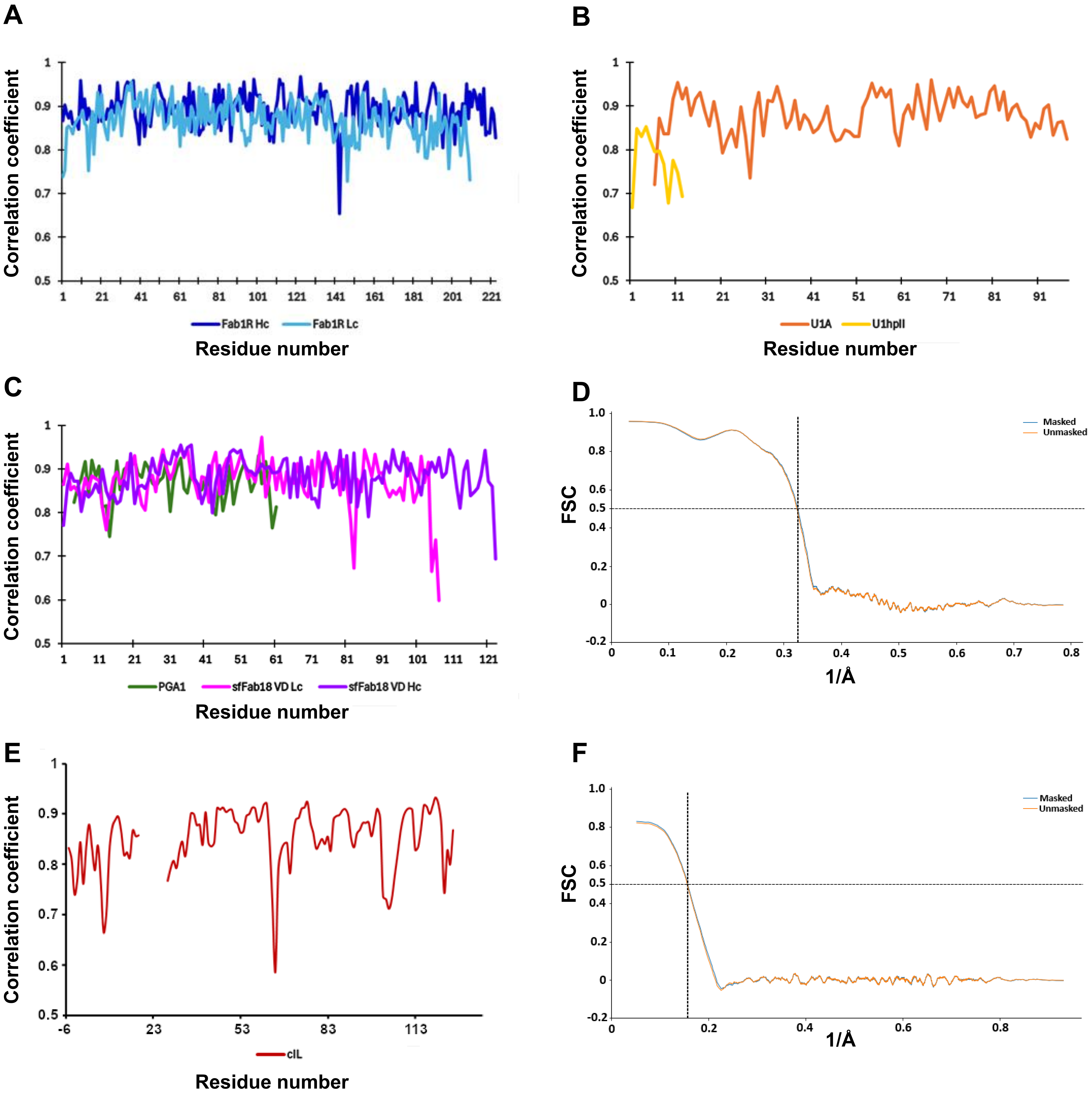

Supplementary Figure S7. Map-to-model comparison of the core and RNA regions. Correlation coefficients are shown for residues of the Fab1R heavy and light chains (**A**); U1A and U1hpII (**B**); PGA1 and the sfFab18 heavy and light chains (**C**); and for nucleotides of the cIL (**E**). The Fourier shell correlation curves for the core (**D**) and RNA region (**F**) are calculated from the model-derived maps with and without masking in *Phenix*. The intersections of the curves with FSC = 0.5 are indicated.

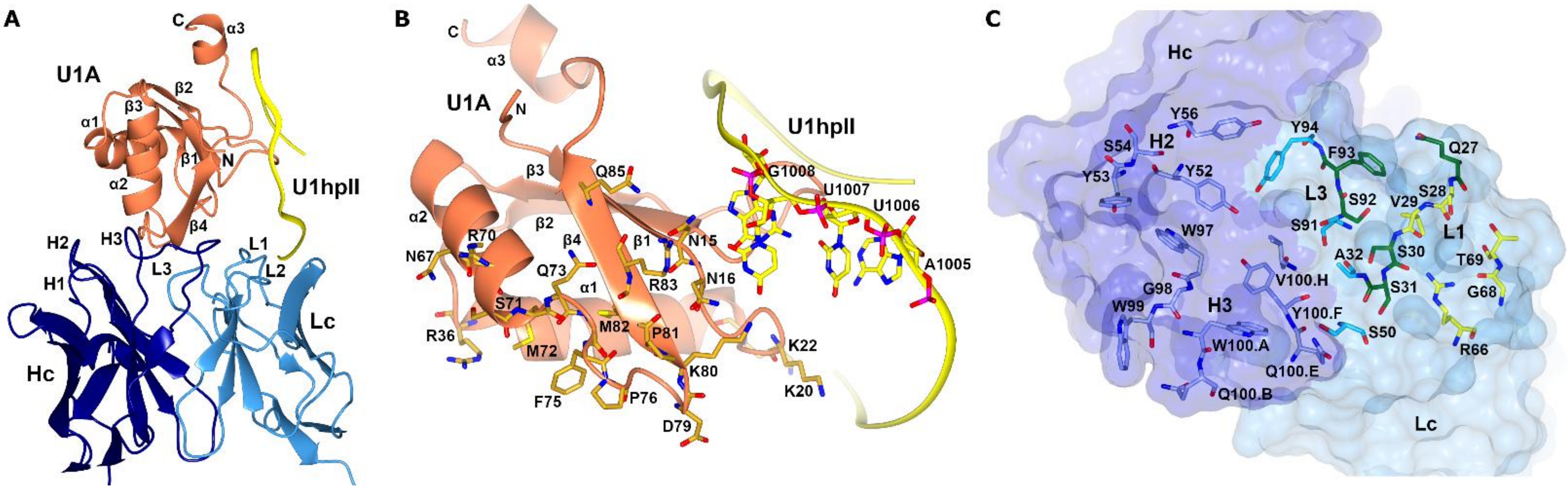

Supplementary Figure S8. Detailed view of the interaction interface among U1A, U1hpII, and Fab1R. (**A**) Schematic depiction of the U1A-U1hpII-Fab1R interface within the core structure of the complex. Secondary structure elements of U1A and the CDRs of Fab1R are indicated. (**B**) Ribbon depiction of U1A and U1hpII highlighting the amino acid residues and nucleotides involved in interactions with Fab1R. (**C**) Surface representation of Fab1R variable domains highlighting amino acid residues contacting U1A and U1hpII loop. Fab1R heavy chain residues contacting U1A are shown in ice blue, while light chain residues interacting with U1A are shown in cyan. Residues interacting with both U1A and U1hpII are in green, and those interacting only with U1hpII are in yellow. Residues are presented as cylinder models, with oxygens in red, nitrogens in blue, and phosphorus atoms in pink.

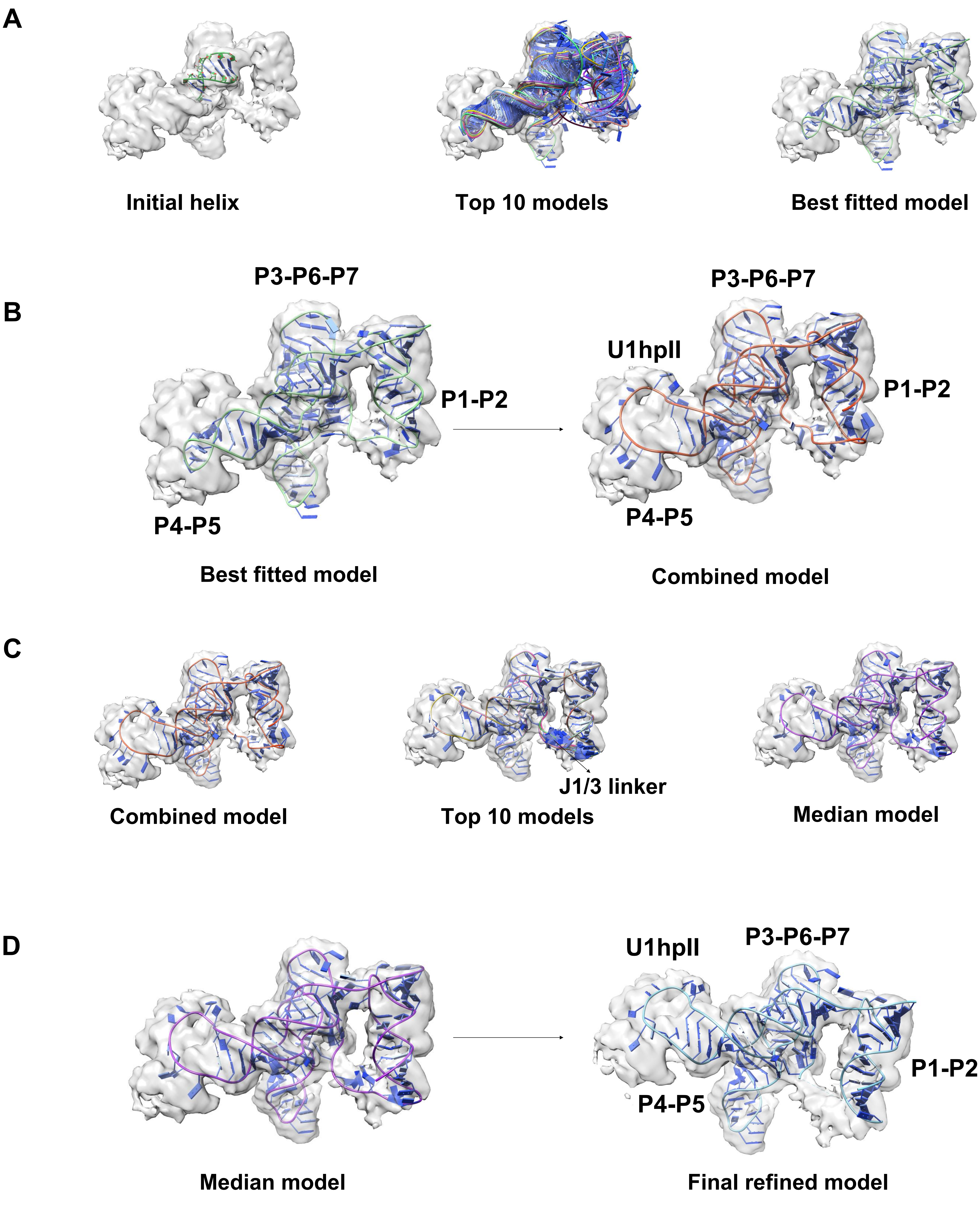

Supplementary Figure S9. Schematic of the auto-DRRAFTER model-building workflow for cIL. (**A**) A single P4 helix was placed into the density map to initiate automated model building, generating the top ten scoring models. (**B**) The catalytic domains of cIL (P3-P6-P7 and P4-P5), including the engineered U1hpII loop, were substituted from the crystal structure and combined with the substrate domain (P1-P2) from the best-fitting model. (**C**) This combined model was then used as the starting structure for a semi-automated round of RNA model building with option that allows to move model within the density map. (**D**) The median model was refined in *Phenix* and *ERRASER* to produce the final structure.

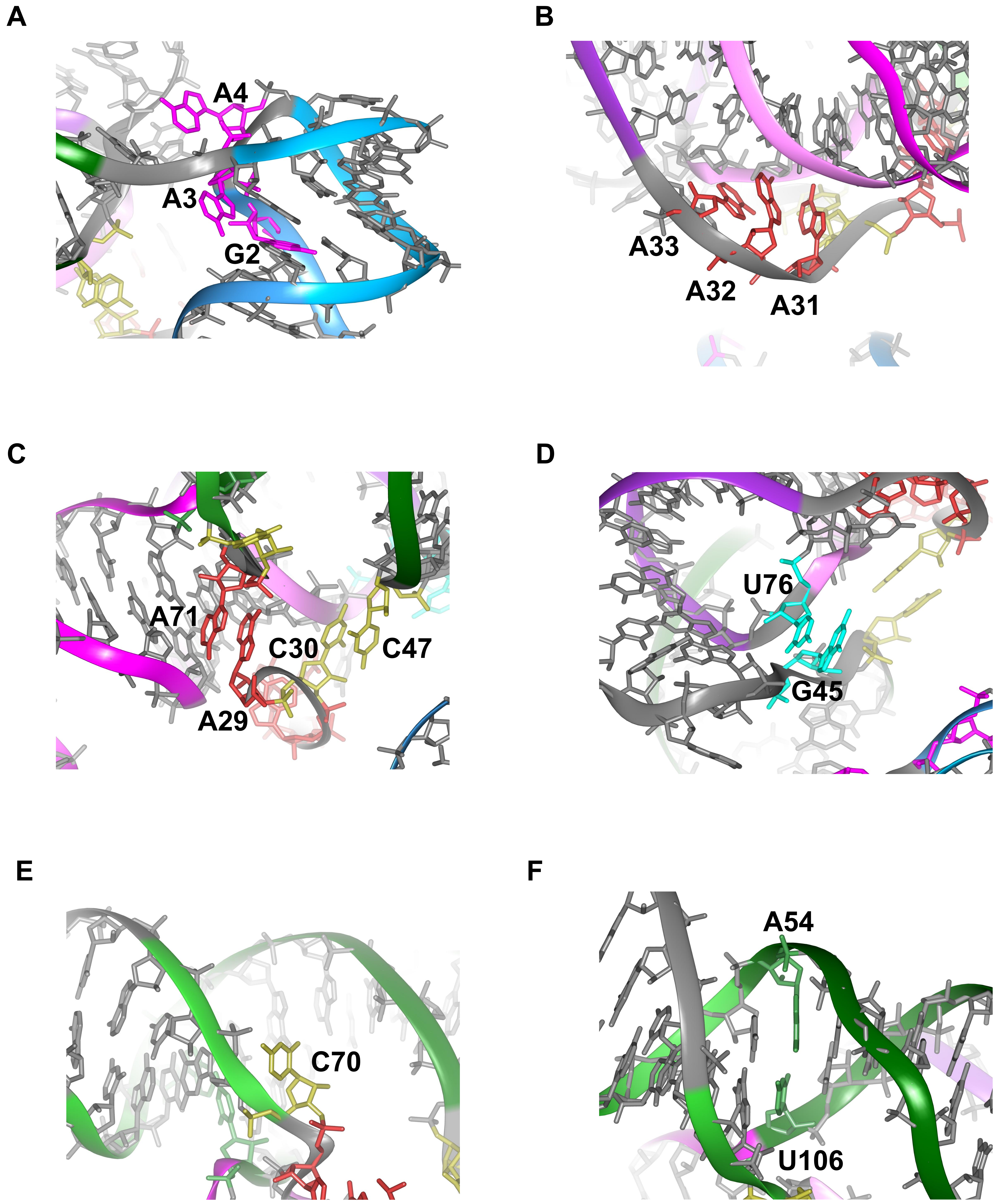

Supplementary Figure S10. Visualization of J1/2 linker nucleotides (**A**) and catalytic-domain nucleotides (**B**-**F**) participating in ternary interactions within the cryo-EM cIL "open" complex.

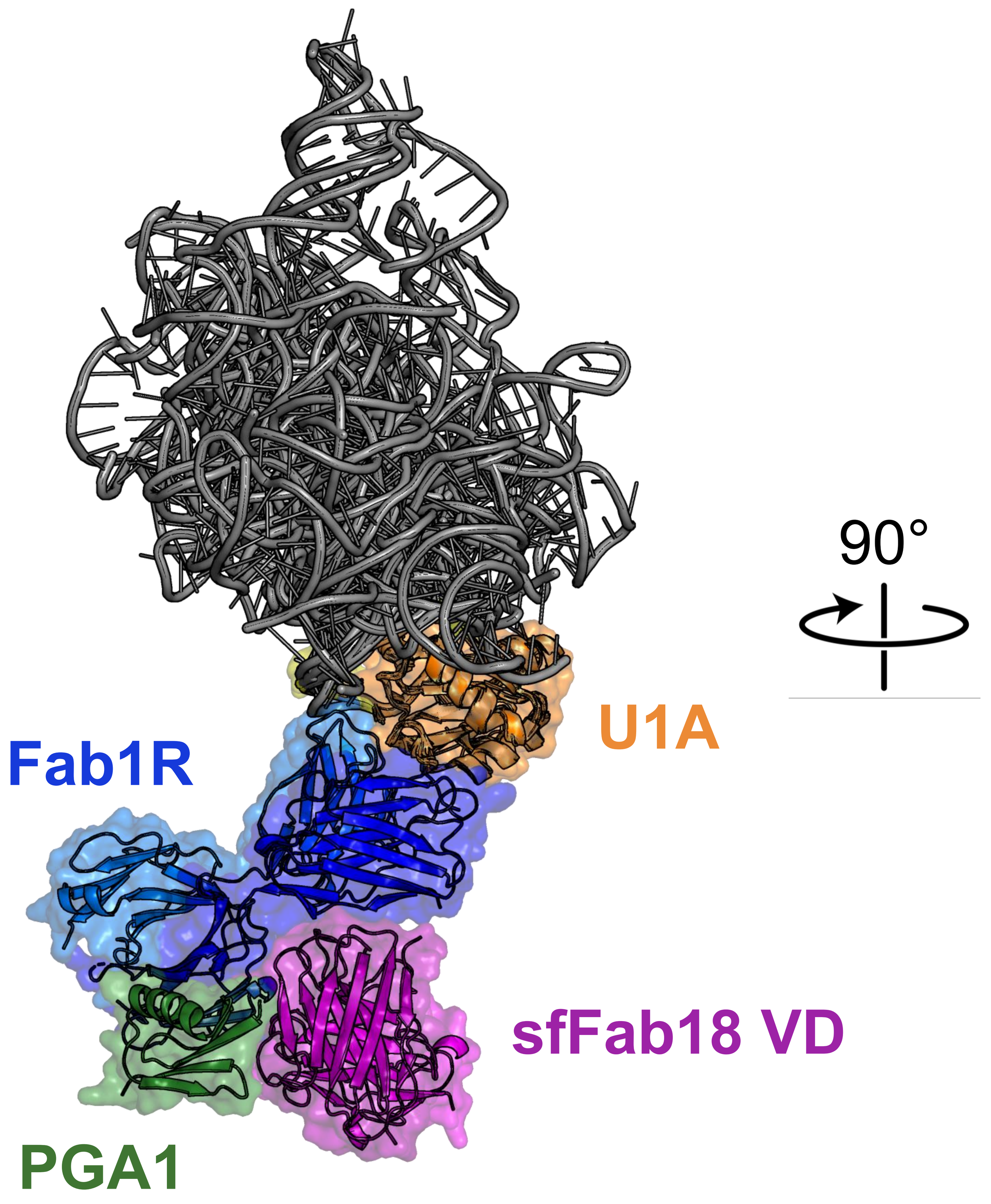

Supplementary Figure S11. Structural superposition of the U1hpII-U1A-Fab1R-PGA-sfFab18 complex onto 20 RNA structures solved using the U1hpII-U1A crystallization module, demonstrating that the complex does not sterically clash with any of these previously characterized RNAs. The alignment was performed on the U1hpII-U1A portion common to all structures. PDB entries included in this analysis: *glmS* ribozyme (3G9C), HDV ribozyme (1DRZ), class I ligase ribozyme (3HHN), TPP riboswitch (3K0J), hairpin ribozyme (1M5P), aminoacyl-tRNA synthetase ribozyme (3CUL), c-di-GMP riboswitch (3IWN, 4YB1), group I intron (1U6B), tetracycline-binding aptamer (3EGZ), glutamine riboswitch (5DDO, 5DDP), NAD^+ riboswitch (7D7V), NAD^+-II riboswitch (8GXB), HmKt-7 k-turn motif (5FJ4), *ydaO* riboswitch (4W90), glycine riboswitch (3P49), SAM-VI riboswitch (6LAS), methyltransferase ribozyme (7DWH), and RhoBAST aptamer (8JY0).
